# A first-in-class inhibitor of homologous recombination DNA repair counteracts tumour growth, metastasis and therapeutic resistance in pancreatic cancer

**DOI:** 10.1186/s13046-025-03389-5

**Published:** 2025-04-24

**Authors:** Juliana Calheiros, Rita Silva, Filipa Barbosa, João Morais, Sara Reis Moura, Sofia Almeida, Elena Fiorini, Silva Mulhovo, Tatiana Q. Aguiar, Tao Wang, Sara Ricardo, Maria Inês Almeida, Lucília Domingues, Sonia A. Melo, Vincenzo Corbo, Maria-José U. Ferreira, Lucília Saraiva

**Affiliations:** 1 LAQV/REQUIMTE, Laboratório de Microbiologia, Departamento de Ciências Biológicas, Faculdade de Farmácia, Universidade do Porto, Rua de Jorge Viterbo Ferreira 228, Porto, 4050-313 Portugal; 2 Research Institute for Medicines (iMed.ULisboa), Faculty of Pharmacy, Universidade de Lisboa, Av. Prof. Gama Pinto, Lisboa, 1649-003 Portugal; 3 Instituto de Ciências Biomédicas Abel Salazar (ICBAS), Universidade do Porto, Rua de Jorge Viterbo Ferreira 228, Porto, 4050-313 Portugal; 4 Institute for Research and Innovation in Health (i3S), Universidade do Porto, Rua Alfredo Allen, 4200-135 Porto, Portugal; 5 Department of Engineering for Innovation Medicine (DIMI), University of Verona, 37134 Verona, Italy; 6 Centro de Estudos Moçambicanos e de Etnociências (CEMEC), Faculty of Natural Sciences and Mathematics, Pedagogical University, Maputo, 21402161 Mozambique; 7 CEB – Centre of Biological Engineering, University of Minho, Braga, 4710-057 Portugal; 8 LABBELS – Associate Laboratory, Braga/Guimarães, Portugal; 9 Institute of Medicine and Pharmacy, Qiqihar Medical University, Qiqihar, Heilongjiang 161006 China; 10 Associate Laboratory i4HB - Institute for Health and Bioeconomy and UCIBIO - Applied Molecular Biosciences Unit, Toxicologic Pathology Research Laboratory, University Institute of Health Sciences (1H-TOXRUN, IUCS-CESPU), Gandra, 4585-116 Portugal; 11 Department of Pathology, Faculty of Medicine University of Porto, Al. Prof. Hernâni Monteiro, Porto, 4200-319 Portugal; 12 Porto Comprehensive Cancer Centre (P.CCC) Raquel Seruca, Porto, Portugal

**Keywords:** Indole alkaloid BBIT20, Anticancer agent, PDAC, DNA damage repair, BRCA1-BARD1 interaction inhibitor

## Abstract

**Background:**

Pancreatic ductal adenocarcinoma (PDAC) is among the cancer types with poorest prognosis and survival rates primarily due to resistance to standard-of-care therapies, including gemcitabine (GEM) and olaparib. Particularly, wild-type (wt)BRCA tumours, the most prevalent in PDAC, are more resistant to DNA-targeting agents like olaparib, restraining their clinical application. Recently, we disclosed a monoterpene indole alkaloid derivative (BBIT20) as a new inhibitor of homologous recombination (HR) DNA repair with anticancer activity in breast and ovarian cancer. Since inhibition of DNA repair enhances the sensitivity of cancer cells to chemotherapy, we aimed to investigate the anticancer potential of BBIT20 against PDAC, particularly carrying wtBRCA.

**Methods:**

In vitro and in vivo PDAC models, particularly human cell lines (including GEM-resistant PDAC cells), patient-derived organoids and xenograft mice of PDAC were used to evaluate the anticancer potential of BBIT20, alone and in combination with GEM or olaparib. Disruption of the BRCA1-BARD1 interaction by BBIT20 was assessed by co-immunoprecipitation, immunofluorescence and yeast two-hybrid assay.

**Results:**

The potent antiproliferative activity of BBIT20, superior to olaparib, was demonstrated in PDAC cells regardless of BRCA status, by inducing cell cycle arrest, apoptosis, and DNA damage, while downregulating HR. The disruption of DNA double-strand breaks repair by BBIT20 was further reinforced by non-homologous end joining (NHEJ) suppression. The inhibition of BRCA1-BARD1 heterodimer by BBIT20 was demonstrated in PDAC cells and confirmed in a yeast two-hybrid assay. In GEM-resistant PDAC cells, BBIT20 showed potent antiproliferative, anti-migratory and anti-invasive activity, overcoming GEM resistance by inhibiting the multidrug resistance P-glycoprotein, upregulating the intracellular GEM-transporter ENT1, and downregulating GEM resistance-related microRNA-20a and GEM metabolism enzymes as RRM1/2. Furthermore, BBIT20 did not induce resistance in PDAC cells. It inhibited the growth of patient-derived PDAC organoids, by inducing apoptosis, repressing HR, and potentiating olaparib and GEM cytotoxicity. The enhancement of olaparib antitumor activity by BBIT20 was confirmed in xenograft mice of PDAC. Notably, it hindered tumour growth and liver metastasis formation, improving survival of orthotopic xenograft mice of PDAC. Furthermore, its potential as a stroma-targeting agent, reducing fibrotic extracellular matrix and overcoming desmoplasia, associated with an enhancement of immune cell response by depleting PD-L1 expression in tumour tissues, renders BBIT20 even more appealing for combination therapy, particularly with immunotherapy.

**Conclusion:**

These findings underscore the great potential of BBIT20 as a novel multifaceted anticancer drug candidate for PDAC treatment.

**Supplementary Information:**

The online version contains supplementary material available at 10.1186/s13046-025-03389-5.

## Introduction

Pancreatic ductal adenocarcinoma (PDAC) is among the most lethal and hard-to-treat cancer types worldwide [1]. The most effective curative approach is surgical resection followed by adjuvant chemotherapy. However, 80–90% of PDAC patients are diagnosed with locally advanced, non-resectable tumours or metastases, contributing to the dismal five-year survival lower than 8% [2–4]. Systemic chemotherapy, primarily gemcitabine (GEM), either alone or in combination therapy with radiotherapy, has been used as the first-line treatment for non-resectable or borderline-resectable PDAC [3, 5, 6]. GEM has become the standard chemotherapy for PDAC, offering only a modest mean survival benefit of two to three months [7]. Compared to GEM alone, the polychemotherapeutic FOLFIRINOX (leucovorin/folinic acid, 5-fluorouracil, irinotecan, and oxaliplatin) nearly doubles median survival in patients with metastatic disease [8]. Additionally, the combination of GEM with nanoparticle albumin-bound paclitaxel (nab-paclitaxel) has been shown to significantly enhance overall survival [9]. Nevertheless, these therapeutic regimens are often associated with drug resistance and severe side effects [10].

Over 15% of PDAC patients harbour mutations in DNA damage response genes, such as *BRCA*, resulting in a homologous recombination (HR) deficiency phenotype [11, 12]. Tumours with BRCA deficiencies demonstrate enhanced sensitivity to DNA-damaging agents, particularly platinum-based chemotherapies [13, 14]. Furthermore, poly(ADP-ribose) polymerase (PARP) inhibitors (PARPi) show promise in treating BRCA-defective tumours, as the simultaneous dysfunction of PARP and BRCA induces synthetic lethality [15]. In fact, in PDAC, PARPi have exhibited therapeutic potential both as monotherapies and in combination with standard treatments like GEM and platinum therapies [16–19]. Accordingly, the PARPi olaparib (OLAP) was approved as maintenance therapy for metastatic germline mutant (mut)BRCA PDAC [20]. However, PARPi therapy faces significant challenges, including acquired resistance, adverse side effects, and lack of efficacy in wild-type (wt)BRCA PDAC [21]. Future directions have been focused on combining PARPi with other DNA repair inhibitors to enhance therapeutic outcomes [22]. Despite its potential, drug resistance is commonly observed [1]. Mechanisms underlying chemoresistance in PDAC include reduced drug uptake, altered drug targets, modified cell cycle checkpoints, enhanced DNA repair, and the influence of microRNAs (miRs) targeting genes associated with drug resistance, proliferation, invasion, metastasis, cell cycle regulation, and apoptosis [23]. Therefore, innovative therapeutic strategies to counteract PDAC chemoresistance are urgently needed.

**Fig. 1:**
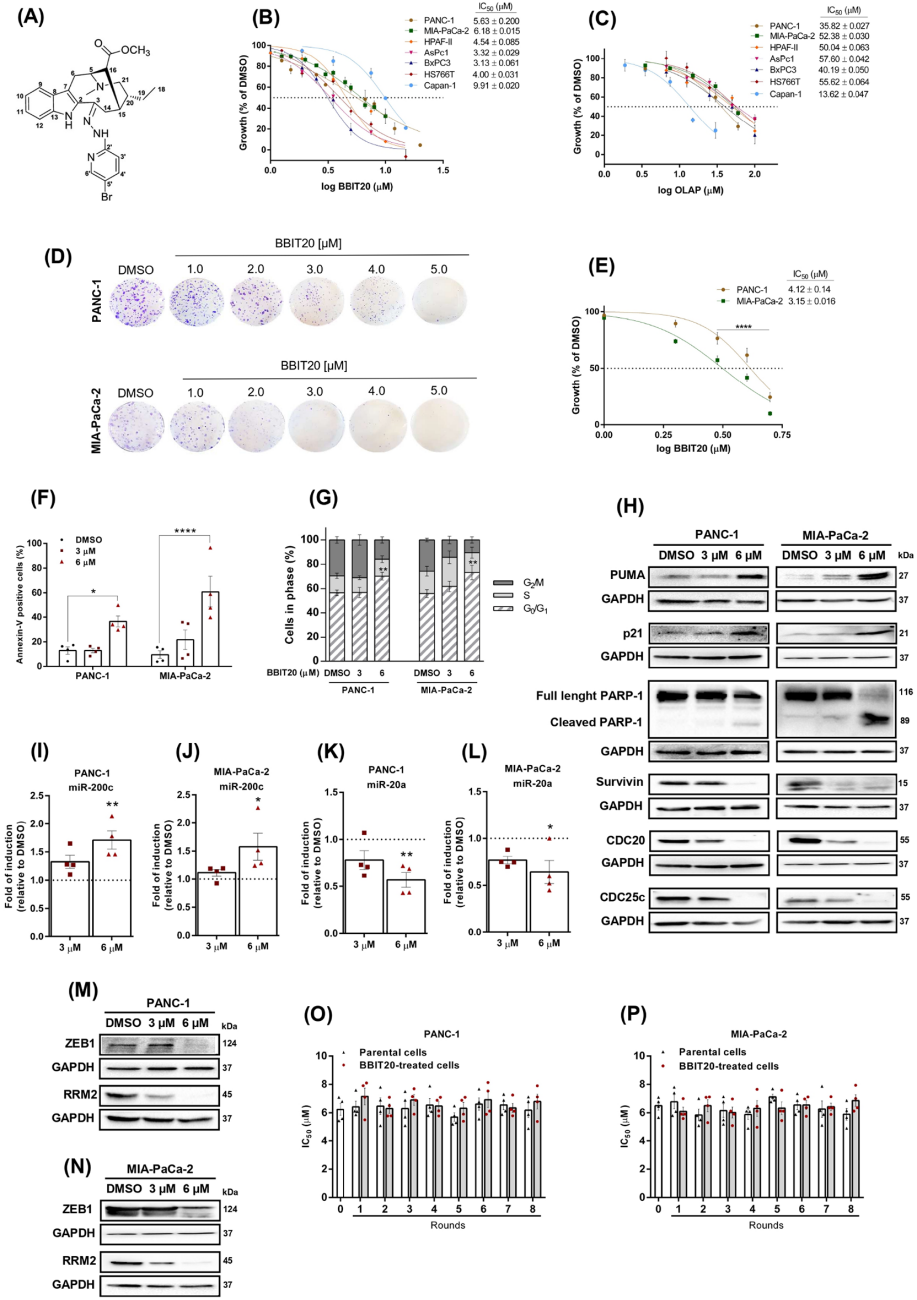
BBIT20 inhibits the growth of PDAC cells by promoting apoptosis and cell cycle arrest, and regulating miRNAs expression levels, without induction of cell resistance. (**A**) Chemical structure of dregamine 5-bromo-pyridin-2-ylhydrazone (BBIT20). (**B**,**C**) Dose-response curves for (**B**) BBIT20 and (**C**) OLAP, in PDAC cells, determined after 48 h of treatment; data are mean ± SEM of four to six independent experiments (two replicates each). (**D**, **E**) Effect of BBIT20 on colony formation of PANC-1 and MIA-PaCa-2 cells, after 15 days of treatment. In (**D**), representative experiments are shown. In (**E**), quantification of colony formation; data are mean ± SEM of four independent experiments, with growth obtained with DMSO set as 100%; growth significantly different from DMSO: ****p < 0.0001 (two-way ANOVA with Sidak’s test). In (**F**-**G**), effect of 3 and 6 µM of BBIT20 on (**F**) apoptosis and (**G**) cell cycle progression of PANC-1 and MIA-PaCa-2 cells, after 48 h of treatment; data are mean ± SEM of four independent experiments; values significantly different from DMSO: *p < 0.05, **p < 0.01, ****p < 0.0001 (two-way ANOVA with Dunnett’s test). In (**H**), expression levels of apoptotic and cell cycle regulators, in PANC-1 and MIA-PaCa-2 cells, after 48 h treatment with 3 and 6 µM of BBIT20. Representative immunoblots are shown; GAPDH was used as a loading control. CDC20 and p21 proteins used the same loading control. In (**I**-**L**), effect of 3 and 6 µM of BBIT20 on the expression levels of (**I**) miR-200c and (**K**) miR-20a, in PANC-1 cells, after 24 h of treatment; (**J**) miR-200c after 24 h of treatment and (**L**) miR-20a levels after 48 h of treatment, in MIA-PaCa-2 cells. Data are mean of fold induction relative to DMSO ± SEM of four independent experiments; values significantly different from DMSO: *p < 0.05, **p < 0.01 (one-way ANOVA with Dunnett’s test). In (**M**), representative immunoblots of the protein levels of ZEB1 and RRM2, after 24 h of treatment with 3 and 6 µM of BBIT20, in PANC-1 cells. In (**N**), representative immunoblots of the protein levels of ZEB1 and RRM2, after 24 h and 48 h of treatment, respectively, with 3 and 6 µM of BBIT20, in MIA-PaCa-2 cells. In (**M**) and (**N**), GAPDH was used as a loading control. In (**O**-**P**), (**O**) PANC-1 and (**P**) MIA-PaCa-2 cells exposed to eight rounds of treatment with increasing concentrations (1, 2, 4, 8, 16, 25, 38 and 45 µM) of BBIT20. IC_50_ values of BBIT20 were determined at the end of each round, after 48 h of treatment. Growth obtained with DMSO was set as 100%; data are mean ± SEM of four independent experiments. IC_50_ values of BBIT20-treated cells not significantly different from parental cells: *p* > 0.05 (two-way ANOVA followed by Sidak’s test)

In a previous study, we have identified a derivative of the natural monoterpene indole alkaloid, the dregamine 5-bromo-pyridin-2-ylhydrazone (BBIT20, Fig. 1A), as an inhibitor of HR DNA repair, disrupting the BRCA1-BARD1 interaction, and demonstrating potent anticancer activity against breast and ovarian cancers [24]. BBIT20 exhibited significant anticancer effects on patient-derived cells and xenograft mouse models of ovarian cancer, with minimal toxicity to non-malignant cells, and no detectable side effects in mice [24].

Herein, we disclosed BBIT20 as a novel anticancer drug candidate for PDAC. Alone or in combination regimens, BBIT20 demonstrated to be highly effective in overcoming PDAC therapeutic resistance.

## Methods

### Compounds

BBIT20 was prepared by condensation reaction of the monoterpene indole alkaloid dregamine, isolated from the alkaloid fraction of the methanol extract of the roots of *Tabernaemontana elegans* with 5-bromo-2-hydrazinopyridine as previously described in [25]. BBIT20, olaparib (OLAP, AZD2281; Santa Cruz Biotechnologies, Frilabo, Portugal) and gemcitabine (GEM, Sigma-Aldrich, Sintra, Portugal), were dissolved in DMSO (Sigma-Aldrich, Sintra, Portugal). The solvent (maximum 0.1% (v/v)) was included as control in the in vitro experiments. BBIT20 was dissolved in 5% (v/v) of DMSO (in corn oil) for in vivo studies.

### Human cancer cell lines and culture conditions

The following human pancreatic ductal adenocarcinoma (PDAC) cell lines were used: AsPC-1 and HS766T (metastatic PDAC), which were grown in RPMI-1640 medium with UltraGlutamine (Biowest, VWR, Carnaxide, Portugal) supplemented with 10% (v/v) heat-inactivated fetal bovine serum (FBS, Biowest, VWR, Carnaxide, Portugal); PANC-1, MIA-PaCa-2, MIA-PaCa-2 non-resistant (parental) and GEM-resistant MIA-PaCa-2 (generated and kindly offered by Dr Luigi Sapio [26]), BxPC3 (PDAC) and HPAF-II (metastatic PDAC), which were grown in DMEM high glucose (4.5 g/L glucose) with stable L-glutamine and sodium pyruvate supplemented with 10% (v/v) FBS. Capan-1 cells (metastatic PDAC) were cultured in Iscove Modified Dulbecco Media (IMDM) 20% (v/v) FBS. Cells were grown at 37 ºC in a 5% CO_2_ humidified atmosphere. Cells were routinely tested for mycoplasma infection using the MycoAlert™ PLUS mycoplasma detection kit (Lonza). Additional information about cells can be found in Table S1.

### Cell viability and proliferation assays

The human PDAC cell lines PANC-1, MIA-PaCa-2, MIA-PaCa-2 non-resistant (parental), GEM-resistant MIA-PaCa-2, AsPC-1, BxPC3, HS766T, HPAF-II, and Capan-1 (5.0 × 10^3^) were seeded in 96-well plates and allowed to adhere overnight, followed by treatment with serial compound dilutions, for 48 h. Sulforhodamine B (SRB) assay was performed as described in [27]. Briefly, cells were fixed with trichloroacetic acid and stained with SRB. The bound dye was solubilized in 10 mM Tris base and the absorbance was measured at 510 nm using a microplate reader (Biotek Instruments Inc., Synergy MX, USA). Absorbance values were used to calculate the percentage of cell viability relative to untreated control cells and the half-maximal inhibitory concentration (IC_50_) values were determined by fitting the dose-response curves using GraphPad Prism software, Version 7.0 (La Jolla, CA, USA).

For the colony formation assay, PANC-1 and MIA-PaCa-2 cells (1 × 10^3^ cells/well) were seeded in six-well plates and treated at the seeding time with a range of concentrations of BBIT20 for 15 days. Colonies were fixed with 10% (v/v) methanol and 10% (v/v) acetic acid for 10 min and stained with 0.5% (w/v) crystal violet (Sigma-Aldrich, Sintra, Portugal) in 1:1 (v/v) methanol/H_2_O for 15 min. Colonies containing more than 20 cells were counted, as performed in [27].

### Cell cycle and apoptosis analysis

PANC-1 and MIA-PaCa-2 cells (1.5 × 10^5^ cells/well) were seeded in six-well plates and allowed to adhere overnight, followed by treatment with BBIT20 for 48 h. Cell cycle and apoptosis analysis were performed as in [27]. Briefly, cells were stained with propidium iodide (Sigma-Aldrich, Sintra, Portugal) and analysed by flow cytometry for the identification and quantification of cell cycle phases. For apoptosis analysis, cells were stained using propidium iodide and Annexin V-FITC Apoptosis Detection Kit I from BD Biosciences (Enzifarma, Porto, Portugal), according to the manufacturer’s instructions. The Accuri™ C6 flow cytometer, BD Accuri C6 software (BD Biosciences, Enzifarma, Porto, Portugal) and FlowJo X 10.0.7 software (Treestar, Ashland, OR, USA) were used.

### Western blot analysis

PANC-1, MIA-PaCa-2 and GEM-resistant MIA-PaCa-2 cells (1.5 × 10^5^ cells/well) were seeded in six-well plates, allowed to adhere overnight, and treated with BBIT20 for 24–48 h. Protein sample preparation and western blot were performed as described in [27]. Briefly, protein lysates were obtained in RIPA buffer with protease inhibitors (Sigma-Aldrich, Sintra, Portugal) and quantified using Pierce^®^ bicinchoninic acid (BCA) protein assay reagents (Thermo Fisher Scientific, Porto Salvo, Portugal). Sodium dodecyl sulphate-polyacrylamide gel electrophoresis (SDS-PAGE) was carried out, and proteins were transferred to a Whatman^®^ nitrocellulose membrane (Amersham Protran, GE Healthcare Life Sciences, Enzymatic, Portugal). Membranes were sectioned to allow the detection of multiple protein targets of distinct molecular weights, blocked with 5% (w/v) skimmed milk or 5% (w/v) bovine serum albumin (BSA) and probed with specific primary and horseradish peroxidase (HRP)-conjugated secondary antibodies (disclosed in Table S2). Glyceraldehyde 3-phosphate dehydrogenase (GAPDH) or vinculin were used as loading controls. Signal was detected with enhanced chemiluminescence (ECL) Amersham (GE Healthcare Life Sciences, Enzymatic, Portugal) using the ChemiDoc™ MP Imaging system (BioRad Laboratories, Amadora, Portugal). Whole blot images are provided in Figure S1. Band intensities were quantified using Image Lab software (version 5.2.1; Bio-Rad laboratories, Amadora, Portugal) and quantification of protein expression levels is represented in Figure S2.

### Acquired resistance studies

PANC-1 and MIA-PaCa-2 cells were exposed to eight rounds of selection with increasing concentrations (1, 2, 4, 8, 16, 25, 38 and 45 µM) of BBIT20, which were added to the culture medium for 24 h, followed by a recovery time of 48 h in fresh medium without treatment. This procedure was previously used to generate doxorubicin-resistant cells [27]. Cells were harvested, seeded and treated twice for each concentration (one round). The same passage number of both parental and resistant cells was used in the experiment. At the end of each round, IC_50_ values were determined by SRB assay, after 48 h of treatment.

### Immunofluorescence

PANC-1 and MIA-PaCa-2 cells (1.5 × 10^4^ cells/well) were seeded in 8-well culture slides (Corning, Thermo Fisher Scientific, Portugal) and allowed to adhere overnight, followed by 48 h treatment with BBIT20. Briefly, cells were fixed in 4% (v/v) paraformaldehyde, permeabilized with 0.5% (v/v) Triton X-100 (diluted in PBS) and blocked in 5% (w/v) BSA (diluted in PBS). Incubation with primary antibodies was performed overnight at 4 ºC, while secondary antibodies were incubated at room temperature for 2 h. Antibodies used are disclosed in Table S2. Nuclei were counterstained with DAPI.

Images were acquired with a Nikon Eclipse C*i* microscope (Nikon, Shinagawa, Tokyo, Japan) equipped with a CoolLed pE-300 lite (CoolLed, Andover, England) and processed with NIS-Elements Imaging software (Nikon, Shinagawa, Tokyo, Japan). Number of foci formation were quantified using ImageJ software [28].

### Non-homologous end joining (NHEJ) reporter assays

PANC-1 cells seeded in 6-well plates were pre-treated for 24 h with either DMSO, 12 µM or 18 µM of BBIT20, then co-transfected using Lipofectamine 3000 reagent with 2 µg EJ5-GFP (addgene, Cat.No. 44026), 2 µg pCBASceI (addgene, Cat.No. 26477), and 2 µg PCI2-HA-mCherry plasmids. After 16 h incubation, the medium was replaced with BBIT20 or DMSO containing medium matching the initial treatment conditions. Following an additional 48 h incubation, cells were analysed by flow cytometry using a FACS Calibur flow cytometer (BD Biosciences, USA), with data processed through FlowJo 10.8.1 software (Treestar, Ashland, OR, USA) to quantify GFP-positive (indicating successful NHEJ repair) and mCherry-positive (transfection control) cell populations. The NHEJ repair efficiency was determined by calculating the ratio of GFP-positive to mCherry-positive cells to normalize for potential variations in transfection efficiency across samples.

### Alkaline COMET assay

PANC-1 and MIA-PaCa-2 cells (1.5 × 10^5^ cells/well) were seeded in six-well plates and allowed to adhere overnight, followed by treatment with 3 and 6 µM of BBIT20 or DMSO for 48 h. DNA damage was evaluated using the OxiSelect Comet Assay Kit (Cell Biolabs, Meditecno, Carcavelos, Portugal) according to the manufacturer’s instructions. Briefly, cells were harvested, resuspended in agarose, spread on slides, and immersed for 40 min in lysis buffer. Electrophoresis was performed in an alkaline electrophoresis solution. Nucleoids were then fixed with 70% (v/v) cold ethanol, stained with Vista Green DNA dye, and photographed using a Nikon DS-5Mc camera and a Nikon Eclipse E400 fluorescence microscope and images were processed with Nikon ACT-2 U software (Izasa Scientific, Carnaxide, Portugal). For each sample, 200 randomly selected nucleoids were quantified using TriTek COMET Score Imaging Software V2.0, measuring the tail DNA (percentage of COMET-positive cells with more than 5% of DNA in the tail) and the tail moment (product of the tail length and percentage of DNA in the tail).

### Co-immunoprecipitation (CO-IP)

PANC-1 and MIA-PaCa-2 cells (5 × 10^5^ cells/flask) were seeded in T25 culture flasks, allowed to adhere overnight, and then treated with 12 and 18 µM of BBIT20. Co-immunoprecipitation was performed after 8 h of treatment with BBIT20 for PANC-1 and after 4 h for MIA-PaCa-2, using the Pierce Classic Magnetic IP and CO-IP Kit as described in [27]. The anti-BRCA1 and anti-immunoglobulin G (IgG) antibodies used for the protein pull-down and the antibodies used for western blot detection of BRCA1, BARD1 and GAPDH are disclosed in Table S2.

### Yeast two-hybrid assay

The full-length wt human *BRCA1* gene was amplified by polymerase chain reaction (PCR) from the plasmid GAL::BRCA1 YEp24 (kind gift from Craig B. Bennett [29]) using the primers Fw_Y2H_BARD1_NdeI (5’ GGAATTCCATATGATGGATTTATCTGCTCTTCGCGTTG) and Rv_BRCA1_SmaI (5’ TCCCCCGGGTCAGTAGTGGCT), and cloned into the *Sma*I-*Nde*I sites of the pGADT7 AD prey vector (Takara Bio, Enzifarma, Porto, Portugal). The full-length *BARD1* gene was amplified by PCR from the plasmid pY3H-AdeI-BARD1 (kind gift from Joanna R. Morris [30]) using the primers Fw_Y2H_BARD1_SmaI (5’ TCCCCCGGGTATGCCGGATAATCGGCAGC) and Rv_BARD1_Notl (5’ ATAAGAATGCGGCCGCATCAGCTGTCAAGAGGAAGC) and cloned into the *Sma*I-*Not*I sites of the pGBKT7 bait vector (Takara Bio, Enzifarma, Porto, Portugal). pGADT7 AD-BRCA1, empty pGADT7 AD and pGADT7-T were transformed into *Saccharomyces cerevisiae* Y187 (Takara Bio, Enzifarma, Porto, Portugal) and selected in SD/-Leu plates. pGBKT7-BARD1 and pGBKT7-53 were transformed into *S. cerevisiae* Y2H Gold (Takara Bio, Enzifarma, Porto, Portugal) and selected in SD/-Leu plates. Following the Matchmaker Gold Yeast Two-Hybrid System User Manual (Takara Bio, Enzifarma, Porto, Portugal), the BARD1 expressing strain was mated with the BRCA1 expressing strain (or strain transformed with the empty pGADT7 AD, to discard bait autoactivation and toxicity) and the diploids were selected on SD/-Trp/-Leu double drop out plates at 30 ºC. Diploids expressing the murine p53 (from pGBKT7-53) and the simian virus 40 (SV40) large tumour (T) antigen (from pGADT7-T) were also generated for use as control.

Diploid cells were grown at 30 ºC and 200 rpm in 5 mL SD/-Trp/-Leu medium until reaching an optical density at 600 nm (OD_600_) of 1. Then, the cells were washed and diluted to an OD_600_ of 0.1 in 1 mL SD/-Leu/-Trp/-His/-Ade medium. In a 96-well plate, 100 µL of these diluted cultures were inoculated in duplicate in 100 µL SD/-Leu/-Trp/-His/-Ade medium containing 0.1% of DMSO (negative control), 10 or 20 µM of BBIT20, to an initial OD_600_ of 0.05. The cultures were then incubated at 30 °C under continuous orbital shaking (200 rpm), until the negative control reached the mid-log phase (OD_600_ of 0.4–0.6). Then, 10-fold serial dilutions were prepared in 0.9% (w/v) NaCl and spotted (5 mL) on SD/-Leu/-Trp/-His/-Ade/X-alpha-Gal plates containing 0.1% DMSO (negative control), 10 or 20 µM of BBIT20. Colony growth and colour were assessed after 2 and 3 days of incubation at 30 ºC. The 10^− 2^ dilution was also plated (50 µL) in triplicate on SD/-Leu/-Trp/-His/-Ade/X-alpha-Gal plates (⌀ 60 mm) containing 0.1% of DMSO (negative control), 10 or 20 µM of BBIT20 for quantification of colony-forming units per liter (CFUs/L) after 2 days of incubation at 30 ºC.

### Proteomic analysis using nanoscale liquid chromatography coupled to tandem mass spectrometry

MIA-PaCa-2 cells (1.5 × 10^5^ cells/well) were seeded in six-well plates, allowed to adhere overnight, and treated with DMSO or 6 µM of BBIT20 for 48 h. Cells were harvested, washed 4× with cold PBS 1× and protein lysates were obtained using RIPA buffer (20 mM Tris-HCl pH 7.5, 150 mM NaCl, 1% (v/v) Triton X-100 and 1% (v/v) NonidetP-40) with protease and phosphatase inhibitors (at 1:100 dilution, Thermo Fisher Scientific, Porto Salvo, Portugal) and quantified with DC Protein Assay (Bio-Rad, Amadora, Portugal). Protein identification and label-free quantitation (100 µg of lysate) was analysed using liquid chromatography-tandem mass spectrometry (nanoLC-MS/MS) as in [31, 32], on an Vanquish Neo liquid chromatography system coupled to a Orbitrap Eclipse™ Tribrid™ mass spectrometer (Thermo Scientific, Porto Salvo, Portugal). Briefly, the extracted protein was loaded onto a trapping cartridge for 3 min and then separated on a nano-C18 column at a flow rate of 300 nL/min. Peptide separation gradient was the following (A: 0.1% (v/v) FA, B: 80% (v/v) ACN 0.1% (v/v)): 5 min (2.5% (v/v) B to 10% (v/v) B), 100 min (10% (v/v) B to 35% (v/v) B), 20 min (35% (v/v) B to 55% (v/v) B), 3 min (55% (v/v) B to 99% (v/v) B) and 12 min (hold 99% (v/v) B). Data acquisition was conducted in a data-dependent acquisition mode controlled by Xcalibur and Tune software (Thermo Scientific, Porto Salvo, Portugal). The mass spectrometer was operated in data-dependent positive acquisition mode alternating between a full scan (m/z 380–1580) and subsequent HCD MS/MS of the 10 most intense peaks from full scan. Raw data were processed using Proteome Discoverer 3.0 software (Thermo Scientific, Porto Salvo, Portugal). Peptide identification was performed with Sequest HT search engine against the *Homo sapiens* entries from UniProt database (https://www.uniprot.org/). Mass tolerance was set as 10 ppm for precursors and 0.02 Da for-fragment ions, respectively, with a maximum of two missed cleavage sites allowed. Cysteine carbamidomethylation was set as a constant modification. Oxidation and N-terminal acetylation of methionine were set as variable modifications. Protein and peptide confidence were set to high. The processing node Percolator was enabled with the following settings: maximum delta Cn 0.05; decoy database search target false discovery rate (FDR) at 1%, validation based on q-value. Samples were normalized against to the total peptide signal and quantitative evaluation was performed using pairwise comparisons. Data correction was applied using the Benjamin-Hochberg method.

Bioinformatics and data analysis were conducted on proteins expressed in the three independent experiments, with a minimum of two unique peptides and high protein confidence, after removal of the contaminants. Differentially expressed proteins were considered when *p*-value < 0.05. Differentially expressed proteins were classified into functional categories including biological process, molecular functions and cellular components according to gene ontology (GO) analysis using the Protein Analysis Through Evolutionary Relationships (PANTHER) classification system (version 19.0), and significantly enriched GO terms (*p*-value < 0.05) were determined using Fisher’s Exact Test with FDR correction. The Database for Annotation, Visualization and Integrated Discovery (DAVID, v6.8) was used to perform pathway enrichment analyses with a significance threshold set at *p*-value < 0.05. The Kyoto Encyclopedia of Genes and Genomes (KEGG) pathway database within DAVID identified significantly enriched pathways, assessed using the EASE score (a modified Fisher Exact *p*-value) with a significance threshold set at *p*-value < 0.05. Protein interaction networks were analysed using the Search Tool for the Retrieval of Interacting Genes/Proteins (STRING) data base version 12.0, with a selection of confidence score threshold of 0.7 (high confidence) and K-means clustering, for interactions. Assessment of the impact on *Disease and Functions* was performed using the software Ingenuity Pathway Analysis (IPA).

### P-glycoprotein (P-gp) activity

GEM-resistant MIA-PaCa-2 cells (3.5 × 10^4^ cells/well) were cultured overnight in a 96-well plate. After 24 h, with approximately 80–90% of cellular confluence, the assay proceeded following the manufacturer’s instructions of the multidrug efflux transporter P-gp ligand screening kit (Abcam, Cambridge, United Kingdom, ab284553). Briefly, cells were incubated with either vehicle (1% DMSO, negative control), 100 µM of verapamil (positive control) or 6 µM of BBIT20 and exposed to P-gp substrate for 30 min to measure the fluorescence intensity (Excitation/Emission = 488/532 nm) or to analyse the intracellular accumulation of the fluorogenic P-gp substrate hydrolysis product by fluorescence microscopy (using the Nikon eclipse Ts2R-C-AL microscope with a Nikon LV-TV camera and NIS Elements BR-5.20 software (Nikon Corporation, Shinagawa, Tokyo, Japan)).

### Combination therapy

PANC-1, MIA-PaCa-2 and GEM-resistant MIA-PaCa-2 cells (5.0 × 10^3^ cells/well) were seeded overnight in 96-well plates and then treated with DMSO (control), BBIT20 (at a concentration with no significant effect on cell growth, IC_10_) and/or increasing concentrations of OLAP or GEM, for 48 h. Monotherapy treatments were included as controls. The effect on cell proliferation was analysed by SRB assay. Mutually nonexclusive combination index (C.I.) and dose reduction index of the chemotherapeutic (D.R.I.) were determined using CompuSyn software (version 1.0, ComboSyn, Inc., Paramus, NJ, USA), as described in [27, 33]. Synergistic interactions of drugs were indicated as C.I. < 1, antagonist interactions as C.I. > 1.1 and addictive effects as 1 < C.I. < 1.1.

### Migration and invasion assays

Cell migration was analysed by wound healing assay and fluorimetric QCM™ 24-well Chemotaxis Cell Migration Kit (8 μm, Merck Millipore, Algés, Portugal). Cell invasion was analysed using the fluorimetric QCM™ 24-well ECMatrix Cell Invasion Kit (8 μm, Merck Millipore, Algés, Portugal), according to the manufacturer’s instructions.

In the wound healing assay, GEM-resistant MIA-PaCa-2 cells (1 × 10^5^ cells/well) were grown to confluence in 2-well silicone inserts (Ibidi, Enzifarma, Porto, Portugal) and a fixed-width wound was created in the cell monolayer removing the culture-insert. Cells were treated with DMSO or 1.5 µM of BBIT20 in serum-privation media and images of the wound were captured at different time points of treatment (0, 6, 24, 30 and 48 h) until complete closure of the wound, using an inverted NIKON TE 2000-U microscope at 100× magnification with a DXM1200F digital camera and an NIS-Elements microscope imaging software (version 4; Nikon Instruments Inc., Izasa, Carnaxide, Portugal). Wound closure was calculated by subtracting the wound area (measured using ImageJ Software) at the indicated time point of treatment to the wound area at the starting point.

For the Chemotaxis Cell Migration assay and fluorimetric cell invasion assay, 0.5 × 10^6^ cells/mL of GEM-resistant MIA-PaCa-2 cells were prepared in serum-free media and treated with 1.5 µM of BBIT20 or solvent. The prepared cell-treated suspensions were distributed into the upper transwell insert (300 µL/insert), followed by the addition of 500 µL medium containing 10% (v/v) FBS to the lower chamber as a chemoattractant. After 24 h, cells that migrated or invaded through the ECMatrix layer with 8 μm pore membranes were eluted, lysed, and stained with a green fluorescence dye that binds to cellular nucleic acids. In both assays, the number of migrated/invaded cells was proportional to the fluorescence signal measured using the Bio-Tek Synergy HT plate reader (Izasa Scientific, Carnaxide, Portugal), at 480/520 nm (excitation/emission).

### MicroRNA quantification by RT-qPCR

PANC-1, MIA-PaCa-2 and GEM-resistant MIA-PaCa-2 cells (1.5 × 10^5^ cells/well) were seeded in 6-well plates and treated with BBIT20 for 24–48 h. RNA was isolated using TRIzol reagent (Invitrogen, Porto Salvo, Portugal) according to the manufacturer’s instructions. RNA concentration and purity were determined by measuring the absorbance at 260 nm using a NanoDrop Spectrophotometer ND-1000 (Thermo Fisher Scientific, Porto Salvo, Portugal).

For the analysis of miRNAs expression levels, TaqMan microRNA Reverse Transcription Kit (Alfagene, Carcavelos, Portugal) and gene specific stem-loop reverse transcription primers (hsa-miR-200c-3p and hsa-miR-20a-5p (Invitrogen, Porto Salvo, Portugal)) were used according to the manufacturer’s instructions. Reverse transcription was performed to synthesize the complementary DNA (cDNA) from total RNA and the qPCR reaction was performed in a CFX Real-Time PCR Detection System (Bio-Rad, Amadora, Portugal), as described in [31]. Small nuclear RNA U6 was used as a reference gene and all the RT-qPCR reactions were performed in duplicates. Data were analysed using Bio-Rad CFX Manager software (Bio-Rad, Amadora, Portugal). Relative expression levels were calculated using the quantification cycle (Ct) method, according to Minimum Information for Publication of Quantitative Real-Time PCR Experiments (MIQE) guidelines [34].

### Culture of PDAC patient-derived organoids

Pancreatic cancer tissues were obtained from patients undergoing surgical resection at the University Hospital Trust of Verona. Ethics committee approval was obtained at University of Verona, Italy: approval number 1885 from the Integrated University Hospital Trust (AOUI) Ethics Committee (Comitato Etico Azienda Ospedaliera Universitaria Integrata). Written informed consent from the donors for research use of tissue in this study was obtained prior to acquisition of the specimen. Samples were confirmed to be tumour or normal based on pathological assessment. All experiments were conducted in accordance with relevant guidelines and regulations. PDAC organoid cultures used in this study were established as previously described in [35]. Genetic characterization of patient-derived PDAC organoids is presented in Table S3. To split the culture, organoids were harvested, triturated, centrifuged and the cellular pellet was resuspended in growth factor-reduced, phenol-red free matrigel (Corning, Milan, Italy). The organoids-matrigel suspension (50 µL) was seeded in a 37 ºC prewarmed 24-well or 6-well suspension plates (Greiner Bio-One, Milan, Italy). Once the matrigel was solidified at 37 ºC, complete growth culture media (consisting of advanced DMEM F12 (Gibco, Thermo Fisher Scientific, Milan, Italy), 2 mM GlutaMAX (Gibco), 10 mM HEPES (Gibco), 1 mg/mL Primocin (InvivoGen, Aurogene, Rome, Italy), 10% (v/v) R-spondin 1 conditioned medium, 50% (v/v) Wnt3a-conditioned medium, 100 ng/mL Noggin (PeproTech, Thermo Fisher Scientific, Milan, Italy), 10 nM gastrin (Tocris Bioscience, Bio-Techne, Milan, Italy), 50 ng/mL epidermal growth factor (Gibco), 100 ng/mL fibroblast growth factor 10 (PeproTech), 1× B27 (Gibco), 10 mM nicotinamide (Sigma-Aldrich, Milan, Italy), 1.25 mM N-acetyl-L-cysteine (Sigma-Aldrich) and 500 nM of the TGF-β signalling inhibitor A83-01 (Tocris Bioscience)) was added to each well.

### Determination of cellular viability after drug treatment in PDAC patient-derived organoids

Established organoid cultures were released from matrigel by incubation with a solution of 2 mg/mL dispase I (Gibco, Thermo Fisher Scientific, Milan, Italy) at 37 ºC for 20 min and then enzymatically dissociated into single-cell suspensions using TrypLE (Thermo Fisher Scientific, Milan, Italy) supplemented with dispase I and DNAse I (Sigma-Aldrich, Milan, Italy) for 10 min at 37 ºC. Cells were resuspended at the density of 1000 cells/well in 10% matrigel (in organoid growth media), and 100 µL of the cell suspension was added into each well of a 96-well plate. After 40 h of growth and organoid formation, 50 µL of drugs or vehicle treatment were applied, adding serial dilutions of BBIT20, OLAP and GEM (alone and/or in combination). After 72 h of treatment, organoid viability was evaluated by measuring ATP content through CellTiter-Glo^®^ Luminescent cell viability assay (Promega, Milan, Italy). Luminescence values for the organoid culture viability of the treated conditions were normalized to the control (vehicle) and the IC_50_ values were determined by fitting the dose-response curves using GraphPad Prism software, Version 7.0 (La Jolla, CA, USA).

### Activated caspase-3/7 staining

Single cells were generated from organoids to plate 1000 cells/well in 100 µL of 10% of matrigel (in complete growth culture media) into a 96-well plate. After 2 days of growth and organoid formation, treatment with control (DMSO), BBIT20, OLAP or GEM was applied. After 72 h of treatment, activated caspase-3/7 cells were stained using CellEvent™ Caspase-3/7 green detection reagent (Thermo Fisher Scientific, Milan, Italy) and nuclei counterstained using Hoechst. Pictures were taken using the EVOS™ M7000 Imaging System (Thermo Fisher Scientific, Milan, Italy). The percentage of caspase-3/7-positive cells were quantified using ImageJ.

### Heterotopic and orthotopic xenograft mouse assays

Antitumour assays using heterotopic xenograft mouse models (NSG strain, The Jackson Laboratory) implanted with PANC-1 cells (expressing wtBRCA) were performed as in [24]. Briefly, PANC-1 cells (2.5 × 10^6^) were implanted subcutaneously (in PBS/Matrigel 1:1; Corning, Enzifarma, Porto, Portugal) in the right flank of mice. After 8 days, mice harbouring tumours with approximately 100 mm^3^ were randomized into four experimental groups (8 animals/group). Seven intraperitoneal injections, three times a week, of vehicle (5% DMSO in corn oil), BBIT20 (2 mg/kg), olaparib (50 mg/kg), or the combination of BBIT20 with olaparib, were performed. Tumour volume was measured three times a week, as described in [24].

To establish the orthotopic xenograft model, female and male C57BL/6 Rag2^−/−^ IL2rg^−/−^ mice were surgically implanted with 1.0 × 10^6^ PANC-1 cells in the pancreas, to develop PDAC tumours, as described in [36, 37]. Briefly, mice were anaesthetized by isoflurane inhalation to perform the surgery. After a small incision in the left abdominal flank, pancreas was exposed and placed on a sterile gauze embedded in NaCl 0.9%, cells were slowly injected using a needle attached to a Hamilton syringe and then the peritoneal and skin layers were sequentially closed with PGA sutures (Surgicryl PGA 6–0).

Tumour growth was confirmed by magnetic resonance imaging (MRI) on a 3 Tesla Bruker BioSpec Maxell scanner (Bruker BioSpin, Ettlingen, Germany; running *ParaVision* 360 v3.5 software) equipped with high power gradients (900 mT/m) and a dedicated 30 mm Bruker volume coil for mouse body imaging. PDAC-bearing mice were randomly assigned into two groups (8 mice/group): 2 mg/kg of BBIT20 or vehicle/control (5% DMSO in corn oil). Treatments with BBIT20 or vehicle were administered intraperitoneally for a maximum of 40 days, 3 times a week. Weekly MRI scans were conducted to monitor tumour progression, with a total of four measurements collected through a longitudinal approach. For this, mice were placed in the animal holder under anaesthesia (1.5–2.5% isoflurane in an air-oxygen mixture with 29–30% FiO_2_), heated with a recirculating water blanket, and monitored for rectal temperature (36–37 ºC) and breathing (60–90 BPM). Tumour volume was measured with T2-weighted ^1^H-MRI (turbo-*RARE* pulse sequence, ×8 acceleration factor, 10 number of slices, repetition time TR = 1200 ms, echo time TE = 89 ms, 6 averages, 150 μm isotropic in-plane resolution, 1 mm slice thickness, without inter-slice gap, and 200 × 200 µm^2^ in-plane resolution), acquired in one orientation (axial). Each session lasted up to 30 min/animal. MRI data were processed in ImageJ (U. S. National Institutes of Health, Bethesda, Maryland, USA). For each animal, tumour region was manually delineated on each slice, and the sum of the areas multiplied by the slice thickness to estimate the volume.

Mice were daily monitored with a score sheet for overall health, behaviour, tumour size, and weight loss. Survival was evaluated from the day of tumour cell inoculation until natural death or euthanasia due to reaching severe symptoms, such as low body condition, jaundice and ascites. At the time of euthanasia (cervical dislocation) or natural death, a necropsy was performed to assess tumour and organs (pancreas, liver and lung) weight and size and liver macro-metastases was counted. Kaplan-Meier survival curves were plotted using GraphPad Prism (La Jolla, CA, USA; version 7.0) software and differences between groups were assessed using the log-rank (Mantel-Cox) test. Statistical significance was defined as *p*-value < 0.05. All mice were housed under standard housing conditions at the i3S animal facility, and all animal procedures were reviewed and approved by the i3S Animal Welfare and Ethics Body, and the animal protocol was approved by DGAV “Direção Geral de Alimentação e Veterinária”.

### Haematoxylin and eosin (H&E), masson’s trichrome, Picrosirius red, Terminal deoxynucleotidyl transferase dUTP Nick End Labelling (TUNEL) and immunohistochemistry staining

Patient-derived organoids treated with DMSO or BBIT20 for 72 h, and tumours and organs from in vivo experiment were fixed in 10% formalin, embedded in paraffin, sectioned at 4 μm, and stained with H&E, Masson’s trichrome, Picrosirius red, TUNEL or specific antibodies for immunohistochemistry. Immunohistochemistry staining was performed using the Novolink Polymer Detection System (Leica Biosystems, Carnaxide, Portugal) according to the manufacturer’s instructions. Antigen retrieval was performed by boiling the sections for 20 min in either 10 mM citrate (pH 6.0) or Tris/EDTA (pH 9.0) buffer. Incubation with primary antibodies (disclosed in Table S2) was performed overnight at 4 ºC. TUNEL assay was performed using the In Situ Cell Death Detection Kit Fluorescein (Roche, Sigma-Aldrich, Sintra, Portugal) according to the manufacturer’s instructions, after antigen retrieval in 10 mM citrate buffer (pH 6.0) and with DAPI nuclear counterstain.

Evaluation of 3,3´-diaminobenzidine (DAB) intensity and quantification of positive cells were performed using ImageJ software, as described in [38]. Whole liver sections were digitized on a slide scanner and the area of liver metastases based on mucin 1 (MUC1) staining were quantified and determined using QuPath [39]. The blue-stained collagen density based on Masson’s trichrome staining, and the red-stained collagen density based on Picrosirius Red, were determined in tumour slides using ImageJ, as performed in [40]. Images were acquired using an Olympus CX31 microscope, coupled to a Digital Olympus EP50 camera system (Olympus Life Science, Barcelona, Spain).

### Statistical analysis

The presented data corresponds to mean ± standard error of the mean (SEM) values of at least three independent experiments, statistically analysed using GraphPad Prism (La Jolla, CA, USA; version 7.0) software. Statistical significance between two groups was analysed using student’s *t*-test. For comparison of multiple groups, statistical analysis relative to controls was performed using one-way or two-way ANOVA with post hoc Sidak’s or Dunnett’s multiple comparison tests. Kaplan–Meier survival curves comparison was evaluated by log-rank (Mantel–Cox) test. Statistical significance was set as *p < 0.05, **p < 0.01, ***p < 0.001 and ****p < 0.0001.

## Results

### BBIT20 inhibits the proliferation of PDAC cells by interfering with key molecular pathways related to cell cycle, apoptosis, microtubule motor activity, drug resistance, and DNA repair

The growth inhibitory effects of BBIT20 (Fig. 1B) and OLAP (Fig. 1C) were assessed by sulforhodamine B (SRB) assay across a panel of PDAC cells harbouring either wt- or mutBRCA (supplementary material, Table S1). The half-maximal inhibitory concentration (IC_50_) values confirmed that BBIT20 exhibited comparable potency in wt- and mutBRCA-expressing PDAC cells with IC_50_ values ranging from 3 to 10 µM (Fig. 1B). Conversely, as expected, OLAP showed its greatest inhibitory effect on mutBRCA2-expressing Capan-1 cells (IC_50_ ~ 14 µM), having low antiproliferative effect on wtBRCA-expressing PDAC cells (PANC-1, MIA-PaCa-2, HPAF-II, AsPc1, BxPC3, and HS766T; IC_50_ > 35 µM) (Fig. 1C).

The antiproliferative effect of BBIT20 on PDAC cells was further evaluated by colony formation assay (Fig. 1D and E). Consistently, a pronounced growth inhibitory effect was obtained with BBIT20 after 15 days of treatment, on PANC-1 and MIA-PaCa-2 cells. Altogether, these results corroborated the promising antiproliferative potential of BBIT20, in PDAC cells, particularly when compared to OLAP.

The growth inhibitory effect of BBIT20, at 6 µM (IC_50_), was associated with a significant induction of apoptosis, as measured by annexin V-positive cells (Fig. 1F), and cell cycle arrest at the G0/G1-phase (Fig. 1G), after 48 h of treatment, in PANC-1 and MIA-PaCa-2 cells. Additionally, in these cells, 6 µM of BBIT20 enhanced the expression of pro-apoptotic proteins, including the p53 upregulated modulator of apoptosis (PUMA), the cell cycle regulator p21, and cleaved PARP (Fig. 1H), after 48 h of treatment. Consistently, it also downregulated the anti-apoptotic factors survivin and cell division cycle protein 20 (CDC20) and 25 C (CDC25C) (Fig. 1H), whose elevated levels are associated with disease progression and poor prognosis in PDAC [41–43].

We also examined the potential of BBIT20 to regulate the expression levels of miRNAs associated with PDAC prognosis and/or drug resistance, specifically focusing on miR-200c, miR-20a, miR-21, miR-29c, miR-146a, and let-7d. Among the microRNAs evaluated, miR-200c and miR-20a emerged as the most promising candidates modulated by BBIT20. MiR-200c is a favourable prognostic biomarker in PDAC, with higher expression levels correlating with improved survival in patients undergoing curative resection [44]. Additionally, upregulation of miR-200c inhibits epithelial-to-mesenchymal transition (EMT) and invasion, suggesting a role as a metastasis suppressor during PDAC development, by repressing *ZEB* family and promoting its degradation [44–46]. Consistently, 6 µM of BBIT20 significantly increased miR-200c levels (Fig. 1I and J), also reducing ZEB1 protein expression, after 24 h of treatment, in PANC-1 (Fig. 1M) and MIA-PaCa-2 (Fig. 1N) cells. Conversely, miR-20a has been described as oncogenic in PDAC, being associated with drug resistance, particularly to GEM. Despite the controversy, its association with ribonucleotide reductase regulatory subunit M2 (RRM2), which is involved in GEM resistance, has been previously established in PDAC [47]. In this work, 6 µM of BBIT20 significantly decreased miR-20a and RRM2 expression, in PANC-1 (after 24 h; Fig. 1K and M) and MIA-PaCa-2 (after 48 h; Fig. 1L and N) cells.

We further evaluated whether PDAC cells acquired resistance to BBIT20. For that, a protocol previously used to generate drug resistant cancer cells, namely to doxorubicin [27], was used. However, BBIT20 has not induced resistance in PANC-1 (Fig. 1O) and MIA-PaCa-2 (Fig. 1P) cells, as demonstrated by the maintenance of its IC_50_ values during the several rounds of treatment with increasing concentrations of the compound.

**Fig. 2:**
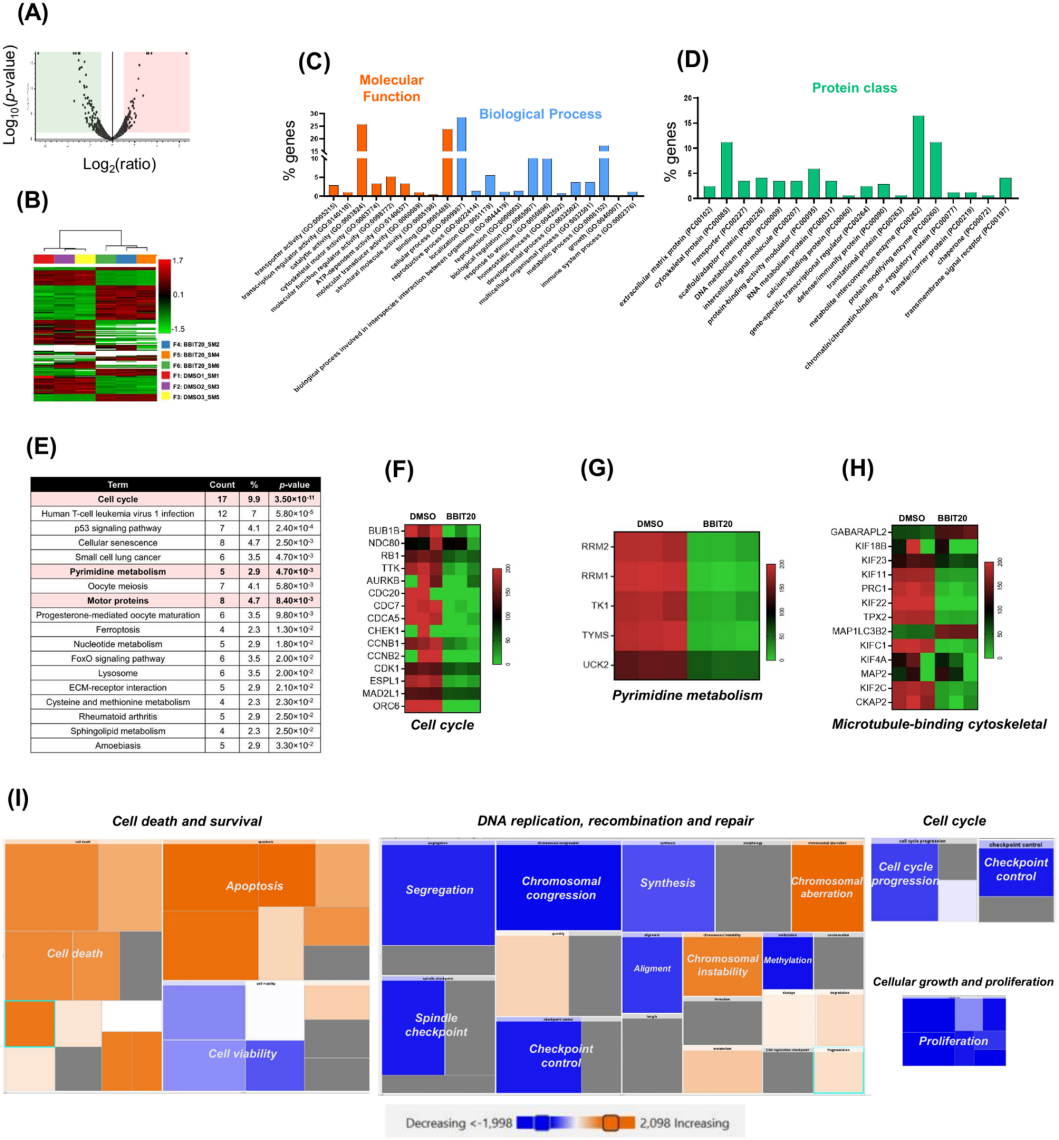
Proteomic analysis identifies downstream targets and molecular pathways triggered by BBIT20, in MIA-PaCa-2 cells. In (**A**), Volcano plot of the differentially expressed proteins, after BBIT20 treatment compared to control group (DMSO). The X-axis represents the log_2_ fold change (FC) and the Y-axis the -log_10_*p*-value. Proteins with *p*-value ≤ 0.05 were considered significant. In (**B**), heat map of the differentially expressed proteins (*p*-value < 0.05), with high confidence false discovery rate (FDR) and at least one sample from each experiment showing detectable protein levels. Sample clustering further illustrates the differential expression patterns (Proteome Discoverer). In (**C**, **D**), bar graphs illustrating the Gene Ontology (GO) terms related to Molecular Functions, Biological Processes, and Protein classes. The analysis was conducted using PANTHER on the 170 differentially expressed proteins (86 downregulated and 84 upregulated). In (**E**), table summarizing the KEGG pathways predicted to be associated with differentially expressed proteins (*p*-value < 0.05), using DAVID Enrichment. In (**F**-**H**), heat maps depicting the differentially expressed proteins (*p*-value < 0.05) associated with specific categories: (**F**) cell cycle, (**G**) pyrimidine metabolism, and (**H**) microtubule-binding cytoskeletal proteins, comparing BBIT20-treated cells to the control group (DMSO). The colour gradient from green to red illustrates the protein expression levels, with green indicating downregulation and red upregulation. In (**I**), tree map of *Disease and Functions* based on the Ingenuity pathway analysis (IPA), highlighting the effects of BBIT20 on critical biological processes such as cell death, survival, DNA replication, recombination and repair, cell cycle, cellular growth and proliferation. The colour gradient from blue to orange represents the activation status of each biological function, with dark blue indicating inhibited functions and dark orange activated functions

To further elucidate the mechanism of action of BBIT20, a proteomic analysis was conducted in a representative wtBRCA PDAC cell line, namely in MIA-PaCa-2 cells, treated with 6 µM of BBIT20 for 48 h. The clustering pattern observed in principal component analysis (PCA) (supplementary material, Figure S3A), volcano plot (Fig. 2A), and heat map (Fig. 2B) confirmed the clear split between BBIT20-treated and control groups, underscoring the consistent response to BBIT20 across the three independent experiments. A total of 5265 proteins were identified and quantified (considering a minimum of two unique peptides and after excluding possible contaminants), in which 170 were differentially expressed based on the following selection criteria: high confidence, *p*-value < 0.05 and consistent expression (increased or decreased) across the different experiments (Supplementary material, Figure S3B and C). Among these 170 differentially expressed proteins, 84 were upregulated and 86 were downregulated (Supplementary material, Figure S3C).

The gene ontology (GO) enrichment analysis, conducted using the PANTHER classification system, systematically categorized the differentially expressed proteins according to their molecular functions, biological processes, and protein classes (Fig. 2C and D). Within the molecular function category, *enzyme regulator activity* (GO:0030234) was particularly prominent, including six proteins related to cell cycle regulation and tumour cell proliferation, which are downregulated following BBIT20 treatment. Although the proliferation marker protein Ki-67 was not associated with any specific PANTHER GO molecular function, it also exhibited a significant downregulation (FC = 0.236; *p*-value = 4.11 × 10^− 6^), after BBIT20 treatment (Supplementary material, Figure S3C). In terms of biological processes, among the 170 differentially expressed proteins, 101 were primarily associated with *cellular processes* (GO: 0009987) and 61 with *metabolic processes* (GO:0008152) (Fig. 2C). The analysis of protein classes indicated that *cytoskeletal proteins* (PC00085) constituted the third largest category of differentially expressed proteins (Fig. 2D). Within this classification, 19 proteins were identified, of which 13 were classified as *microtubule* or *microtubule-binding cytoskeletal proteins*. These data suggested that BBIT20 plays a crucial role in modulating mitotic spindle assembly and the dynamics of the cytoskeleton and microtubules, particularly during cell division (Fig. 2D and H). In addition to the GO enrichment analysis, Kyoto Encyclopedia of Genes and Genomes (KEGG) database was assessed to identify the principal pathways influenced by BBIT20, in MIA-PaCa-2 cells. Upon BBIT20 treatment, 18 pathways were predicted to be affected (*p*-value < 0.05), with the cell cycle being the top-ranked (Fig. 2E). In this pathway, altered proteins were related to cell cycle regulation, mitosis and checkpoint control (Fig. 2F). Furthermore, the KEGG analysis uncovered additional pathways modulated by BBIT20 that are relevant to PDAC, including the p53 signalling pathway (*p*-value = 2.40 × 10^− 4^) (Fig. 2E). Notably, the pyrimidine metabolism pathway (*p*-value = 4.70 × 10^− 3^), which is associated with drug resistance, particularly to GEM, was predicted to be affected (Fig. 2E and G). In fact, proteomic analysis corroborated that BBIT20 downregulated key enzymes involved in GEM metabolism, such as ribonucleotide reductase catalytic subunit M1 (RRM1) (FC = 0.128; *p*-value = 1.08 × 10^− 12^), RRM2 (FC = 0.125; *p*-value = 8.82 × 10^− 16^), and thymidylate synthase (TS/TYMS) (FC = 0.091; *p*-value = 8.82 × 10^− 16^) (Fig. 2G). Notably, RRM2 emerged as one of the most repressed proteins in MIA-PaCa-2 cells treated with BBIT20 (Fig. 2G; Supplementary material, Figure S3C), supporting the marked reduction of RRM2 detected by western blot analysis in BBIT20-treated PANC-1 and MIA-PaCa-2 cells (Fig. 1M and N). Additionally, BBIT20 significantly influenced the expression of motor proteins (*p*-value = 8.40 × 10^− 3^), further reinforcing its impact on mitosis and microtubule dynamics (Fig. 2E and H).

To investigate potential interactions among the differentially expressed proteins, a protein-protein interaction network analysis was performed, in MIA-PaCa-2 cells treated with BBIT20, using the STRING software (Supplementary material, Figure S3D). This analysis revealed four distinct clusters based on high-confidence protein interactions (0.700) and k-means clustering. Notably, cluster 1 comprised 81 proteins, highlighting the inhibitory effects of BBIT20 on pathways associated with genomic stability, particularly those involved in DNA repair and the cellular response to DNA damage (Supplementary material, Figure S3D). Further corroborating our previous data in PANC-1 and MIA-PaCa-2 cells (Fig. 1), the *Disease and Functions* of Ingenuity Pathway Analysis (IPA) predicted that BBIT20 promotes cell death, chromosomal instability and aberration, and decreases cell cycle progression, cell cycle checkpoint control, DNA synthesis, and proliferation (Fig. 2I).

Altogether, the proteomic analysis, in PDAC cells, revealed the ability of BBIT20 to significantly modulate proteins associated with distinct biological mechanisms, including cell cycle, proliferation, microtubule motor activity, drug resistance, and DNA repair.

### BBIT20 disrupts the BRCA1-BARD1 interaction, inhibiting DNA double-strand breaks (DSBs) repair pathways, and inducing genotoxicity in PDAC cells

**Fig. 3:**
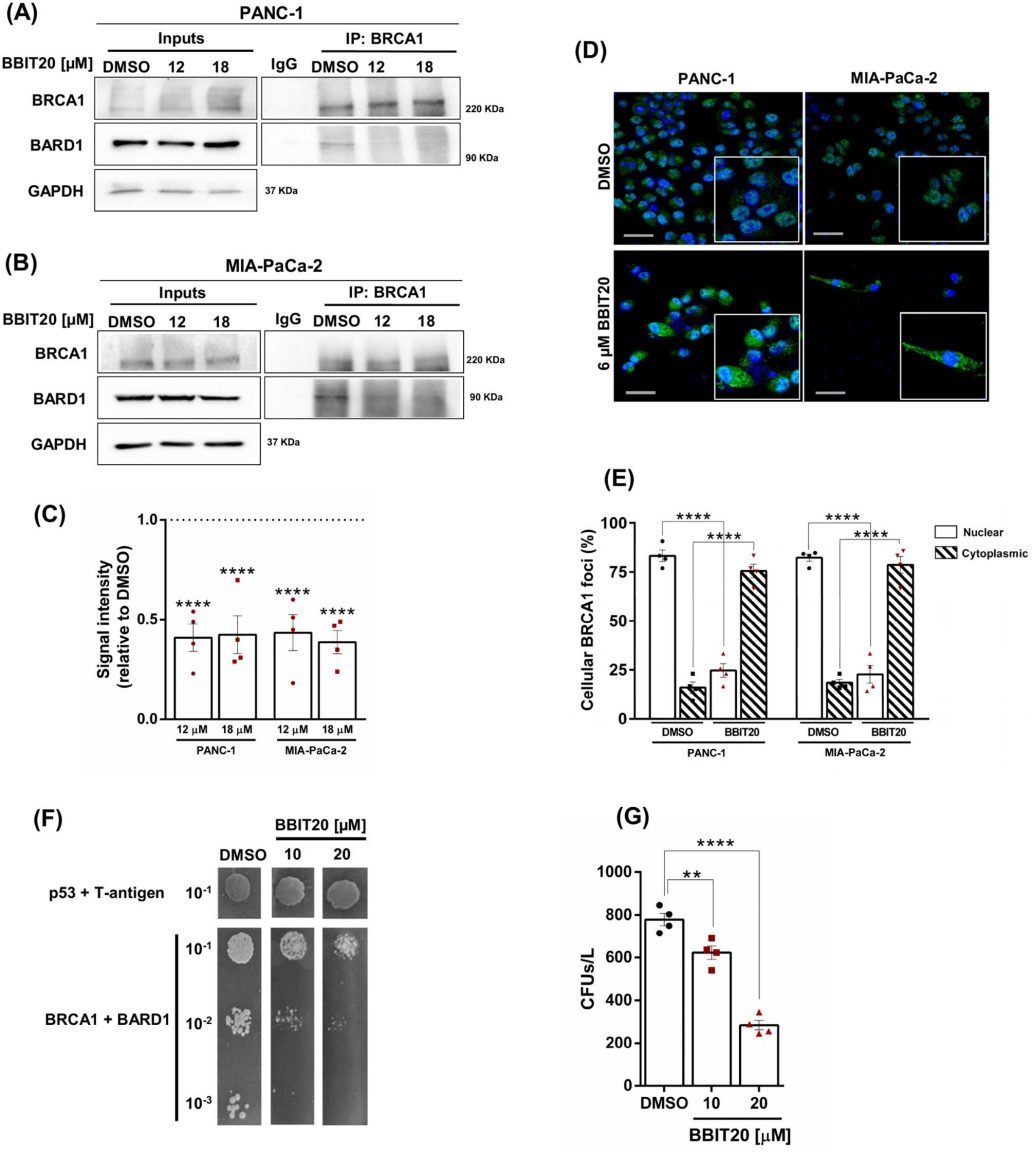
BBIT20 inhibits the BRCA1-BARD1 interaction, in PDAC and yeast cells. (**A**-**C**) Disruption of the BRCA1-BARD1 interaction by 12 and 18 µM of BBIT20, after (A) 8 h (in PANC-1 cells) and (**B**) 4 h (in MIA-PaCa-2 cells), evaluated by co-immunoprecipitation. In (**A**, **B**), representative immunoblots are shown, with whole-cell lysates represented as inputs. GAPDH was used as a loading control for inputs. In (**C**), quantification of BARD1 protein levels co-immunoprecipitated with BRCA1, using BRCA1 from the co-immunoprecipitation as a loading control; values with DMSO were set as 1; data are mean ± SEM of four independent experiments; values significantly different from DMSO: ****p < 0.0001 (two-way ANOVA with Dunnett’s test). In (**D**, **E**), nuclear-cytoplasmic translocation of BRCA1, after 48 h of treatment with 6 µM of BBIT20, analysed by immunofluorescence. In (**D**), representative images (scale bar = 50 μm; 400× magnification) of BRCA1 staining (green) with nuclear counterstaining (DAPI, blue) are shown. In (**E**), quantification of BRCA1 nuclear and cytoplasmic foci formation, represented as mean ± SEM of four independent experiments (100 cells per sample); values significantly different from DMSO: ****p < 0.0001 (two-way ANOVA with Sidak’s test). In (**F**, **G**), yeast two-hybrid assay showing the effect of 10 and 20 µM of BBIT20 on disrupting the BRCA1-BARD1 interaction, evaluated by measuring the growth of diploid yeast strain co-expressing BRCA1 and BARD1 on SD/-Leu/-Trp/-His/-Ade/X-alpha-Gal plates. In (**F**), effect of 10 and 20 µM of BBIT20 on disrupting the BRCA1-BARD1 interaction, but not murine p53-SV40 large T-antigen interaction (control), in diploid yeast strains. Representative images are shown, colony growth was assessed after 2 days of incubation at 30 ºC. In (**G**), quantification of colony-forming units per liter (CFUs/L) of a diploid yeast strain co-expressing BRCA1 and BARD1 treated with 10 and 20 µM of BBIT20, after 2 days of incubation at 30 ºC; data are mean ± SEM of four independent experiments; values significantly different from DMSO: **p < 0.01, ****p < 0.0001 (one-way ANOVA with Dunnett’s test)

Following our previous work in ovarian and breast cancer cells [24], which demonstrated an inhibitory effect of BBIT20 on HR DNA repair by disrupting the BRCA1-BARD1 interaction, we investigated whether BBIT20 also affected the BRCA1-BARD1 interaction, in PDAC cells. For that, a co-immunoprecipitation assay was conducted in MIA-PaCa-2 and PANC-1 cells, after 4 and 8 h treatment with BBIT20, respectively (Fig. 3A-C). Of note, the early time points of 4 and 8 h were selected to avoid a significant decrease in BRCA1 protein levels in the whole cell lysates (which implicated an adjustment of BBIT20 dosage to 2–3 times its IC_50_ value). The results showed a significant reduction of BARD1 protein levels co-immunoprecipitated with BRCA1, confirming the disruption of the BRCA1-BARD1 interaction by 12 and 18 µM of BBIT20 (Fig. 3A-C).

BRCA1 plays its role in association with its binding partner BARD1, which stabilizes and confines BRCA1 at the nucleus to allow HR DNA repair [48]. Disruption of this functional heterodimer leads to BRCA1 translocation to the cytoplasm, thereby compromising HR DNA repair [49]. Consistent with this, we observed by immunofluorescence that 48 h of treatment with 6 µM of BBIT20 triggered BRCA1 cytoplasmic shuttling, in PANC-1 and MIA-PaCa-2 cells (Fig. 3D). This was evidenced by the significant reduction of BRCA1 foci at the nucleus, and a corresponding increase at the cytoplasm (Fig. 3E).

To further validate the inhibitory effect of BBIT20 on the BRCA1-BARD1 interaction, a yeast two-hybrid assay was performed. In the diploid strain expressing the human full-length forms of BRCA1 (prey) and BARD1 (bait), we first confirmed the interaction between these proteins in yeast, as visualized by the pronounced cell growth (colony-forming units, CFUs) due to the activation of auxotrophic reporter genes (DMSO, Fig. 3F and G). An experimental control was also included, testing the known interacting proteins murine p53 and SV40 large T-antigen (DMSO, Fig. 3F). As anticipated, BBIT20 significantly reduced the number of CFUs in a concentration-dependent manner (Fig. 3F and G), confirming the disruption of the BRCA1-BARD1 interaction by the compound. Importantly, BBIT20 has not affected the interaction between p53 and SV40 large T-antigen, as evidenced by comparable growth of yeast treated with BBIT20 and DMSO, which highlighted the selectivity of the compound for the BRCA1-BARD1 interaction (Fig. 3F and G).

**Fig. 4:**
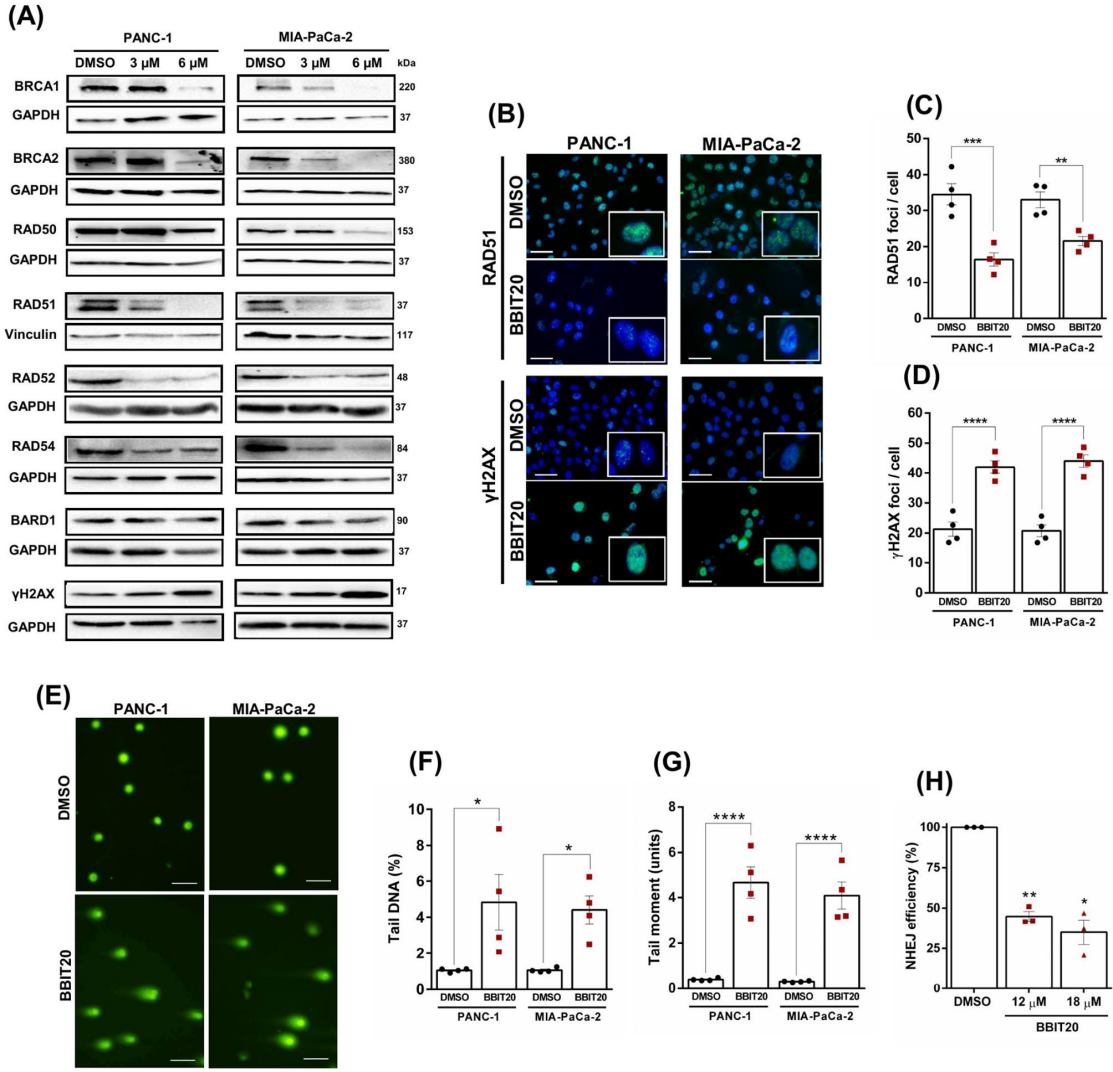
BBIT20 inhibits HR and NHEJ DSBs repair pathways, inducing DNA damage, in PDAC cells. In (**A**), protein levels of BRCA1, BRCA2, RAD50, RAD51, RAD52, RAD54, BARD1 and γH2AX, in PANC-1 and MIA-PaCa-2 cells, after 48 h of treatment with 3 and 6 µM of BBIT20; representative immunoblots of four independent experiments are shown; GAPDH or vinculin were used as loading controls. In (**B**), immunofluorescence of RAD51 and γH2AX foci formation after 48 h of treatment with 6 µM of BBIT20; representative images are shown (scale bar = 50 μm, 400× magnification). In (**C**, **D**), quantification of (**C**) RAD51 and (**D**) γH2AX nuclear foci, in PANC-1 and MIA-PaCa-2 cells. Data are mean ± SEM of four independent experiments (200 cells per sample). Quantification of the number of foci/cell significantly different from DMSO: **p < 0.01, ***p < 0.001, ****p < 0.0001 (two-way ANOVA with Sidak’s test). In (**E**-**G**), evaluation of DNA damage in PANC-1 and MIA-PaCa-2 cells, after 48 h of treatment with 6 µM of BBIT20, by COMET assay. In (**E**), representative images are shown (scale bar = 100 μm, 200× magnification). In (**F**, **G**), quantification of (**F**) tail DNA percentage and (**G**) tail moment; data are mean ± SEM of four independent experiments (200 cells per sample); values significantly different from DMSO: *p < 0.05, ****p < 0.0001 (two-way ANOVA with Dunnett’s test). In (**H**), NHEJ efficiency in PANC-1 treated with DMSO, 12 and 18 µM of BBIT20. Data are mean ± SEM of three independent experiments. NHEJ efficiency was calculated as the ratio of GFP-positive to mCherry-positive cells. Values significantly different from DMSO: *p < 0.05, **p < 0.01 (one-way ANOVA with Dunnett’s test)

In line with this inhibitory effect of BBIT20 on the BRCA1-BARD1 interaction, we verified that 3 and 6 µM of BBIT20 downregulated the expression of crucial proteins involved in HR DNA repair, including BRCA1, BRCA2, RAD50, RAD51, RAD52, and RAD54, in PANC-1 and MIA-PaCa-2 cells, after 48 h of treatment (Fig. 4A). Of note, no significant alterations of BARD1 expression levels were observed upon 3 and 6 µM of BBIT20 treatment (Fig. 4A).

Although key HR proteins were not detected in the proteomic analysis, potentially due to issues related to protein extraction methods and their low natural abundance [50], or were excluded from the analysis for lacking a minimum of two unique peptides, we confirmed their downregulation in BBIT20-treated PANC-1 and MIA-PaCa-2 cells by western blot. Despite this, the proteomic analysis, in MIA-PaCa-2 cells treated with 6 µM of BBIT20 for 48 h, revealed a reduction of RAD51 levels (detected in 2 out of 3 experiments, mean of FC relative to DMSO = 0.421) (Supplementary material, Figure S3E). Accordingly, 6 µM of BBIT20 inhibited nuclear RAD51 foci formation (a surrogate biomarker of HR repair functionality [51]), in PANC-1 and MIA-PaCa-2 cells, after 48 h of treatment (Fig. 4B and C). Altogether, likewise in ovarian and breast cancer cells [24], where we demonstrated potent HR inhibition using an HR reporter assay, also in wtBRCA PDAC cells BBIT20 induced a state of HR deficiency (BRCAness phenotype). Subsequently, we next assessed the level of DNA damage induced by BBIT20, in PANC-1 and MIA-PaCa-2 cells, after 48 h of treatment. As anticipated, BBIT20 increased the levels of phosphorylated gamma histone H2AX (γH2AX) (Fig. 4A), a sensitive marker of DNA double-strand breaks [52]. Consistently, the treatment with 6 µM of BBIT20 resulted in a notable increase in γH2AX foci formation, after 48 h of treatment (Fig. 4B and D). Furthermore, a single-cell level DNA damage was evaluated by the COMET assay (Fig. 4E-G). The results showed that 6 µM of BBIT20 significantly increased the percentage of COMET-positive cells, particularly of tail DNA (Fig. 4F) and tail moment (Fig. 4G), in PANC-1 and MIA-PaCa-2 cells, after 48 h treatment. Overall, these findings confirmed the genotoxic effect of BBIT20 in PDAC cells.

To assess whether BBIT20 could also impact the alternative DSBs repair pathway, non-homologous end joining (NHEJ), we conducted an NHEJ reporter assay. The results revealed that 12 and 18 µM of BBIT20 significantly inhibited NHEJ efficiency in PANC-1 cells (Fig. 4H), further reinforcing a robust inhibition of DSBs repair by BBIT20.

### BBIT20 does not induce cross-resistance in GEM-resistant PDAC cells, sensitizing these cells to GEM and restraining their migration and invasion

**Fig. 5:**
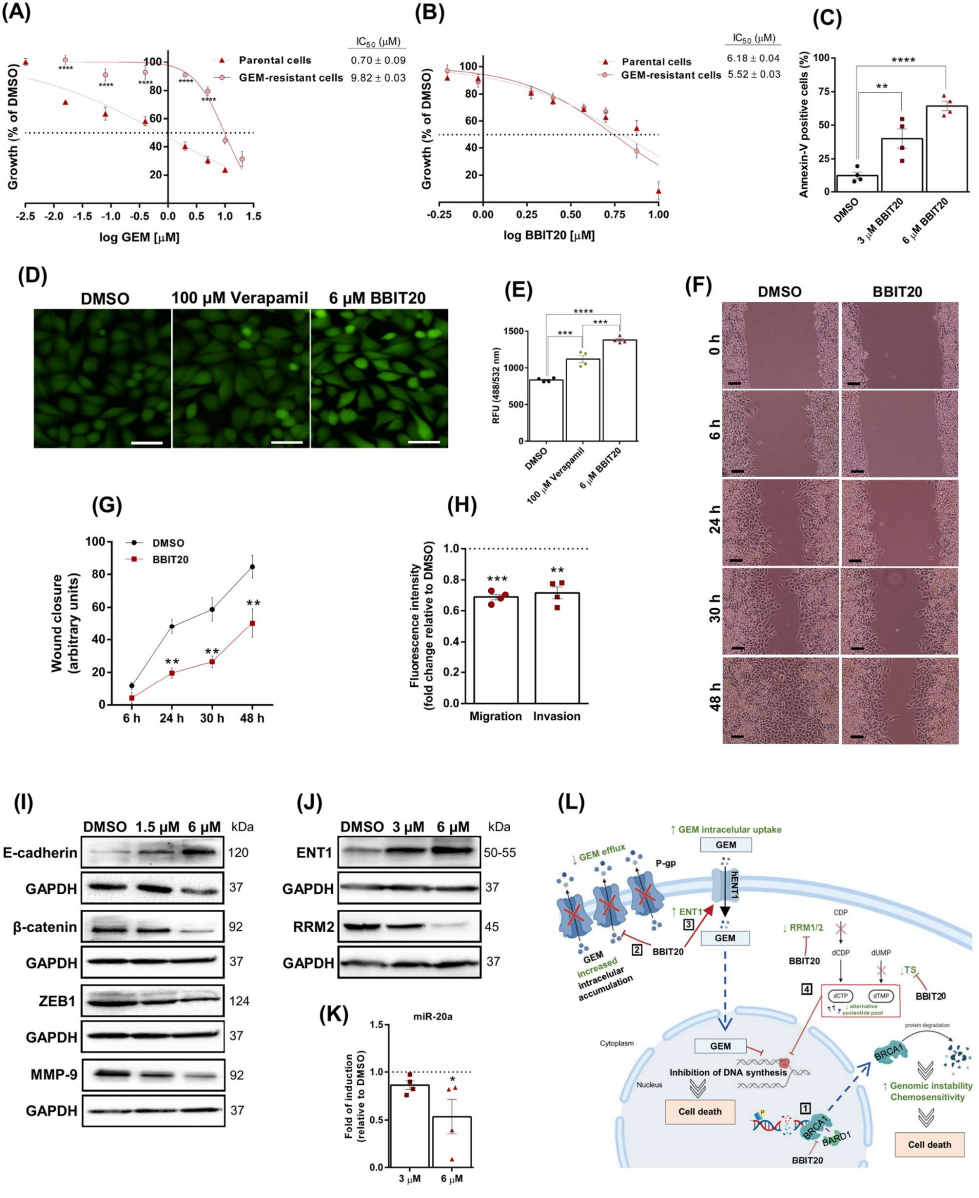
BBIT20 does not induce cross-resistance in GEM-resistant MIA-PaCa-2 cells, inducing apoptosis, inhibiting P-glycoprotein drug efflux, and counteracting migration, invasion, EMT and GEM resistance. In (**A**, **B**), concentration-response curves for (**A**) GEM and (**B**) BBIT20, in non-resistant (parental) and GEM-resistant MIA-PaCa-2 cells, after 48 h of treatment. Growth obtained with control (DMSO) was set as 100%; data shown are mean ± SEM of four independent experiments (two replicates each); values of GEM-resistant cell growth significantly different from parental cells: ****p < 0.0001 (two-way ANOVA with Sidak’s test). In (**C**), effect of 3 and 6 µM of BBIT20 on apoptosis induction in GEM-resistant MIA-PaCa-2 cells, after 48 h of treatment; data are mean ± SEM of four independent experiments; values significantly different from DMSO: **p < 0.01, ****p < 0.0001 (one-way ANOVA with Dunnett’s test). In (**D**, **E**), fluorescence intensity of P-gp activity in GEM-resistant MIA-PaCa-2 cells treated with DMSO (negative control), 100 µM of verapamil (positive control) and 6 µM of BBIT20, using the multidrug efflux transporter P-gp ligand screening kit, after 30 min of treatment. In (**D**), representative fluorescence images (scale bar = 50 μm; 400× magnification) of the intracellular accumulation of the fluorogenic P-gp substrate hydrolysis product, in GEM-resistant MIA-PaCa-2 cells treated with DMSO, 100 µM of verapamil (P-gp inhibitor) or 6 µM of BBIT20. In (**E**), mean of fluorescence intensity (Excitation/Emission = 488/532 nm) ± SEM of four independent experiments; values significantly different from DMSO or verapamil: ***p < 0.001, ****p < 0.0001 (one-way ANOVA with Tukey’s test). In (**F**, **G**), effect of 1.5 µM (IC_10_) of BBIT20 on confluent GEM-resistant MIA-PaCa-2 cell migration after 6, 24, 30 and 48 h of treatment. In (**F**), representative images of the wound healing are shown (scale bar = 100 μm, 100× magnification). In (**G**), quantification of the wound closure determined considering randomly selected microscopic fields (four fields per sample), setting the initial wound area as 100%; data are mean ± SEM of four independent experiments; values significantly different from DMSO: **p < 0.01 (two-way ANOVA with Sidak’s test). In (**H**), effect of 1.5 µM (IC_10_) of BBIT20 on migration and invasion of GEM-resistant MIA-PaCa-2 cells, after 24 h of treatment. Migratory and invasive cells were quantified by fluorescence signal intensity; values obtained with DMSO were set as 1. Data are mean ± SEM of four independent experiments; values significantly different from DMSO: **p < 0.01, ***p < 0.001 (unpaired student’s *t*-test). In (**I**), protein expression levels of E-cadherin, β-catenin, ZEB1 and MMP-9, after 48 h of treatment with 1.5 and 6 µM of BBIT20, in GEM-resistant MIA-PaCa-2 cells; representative immunoblots of four independent experiments are shown; GAPDH was used as a loading control. β-catenin and ZEB1 proteins used the same loading control. In (**J**), effect of 3 and 6 µM of BBIT20 on protein levels of ENT1 and RRM2 after 24 h of treatment, in GEM-resistant MIA-PaCa-2 cells. Representative immunoblots of four independent experiments are shown; GAPDH was used as a loading control. In (**K**), expression levels of miR-20a, after 24 h of treatment, in GEM-resistant MIA-PaCa-2 cells. Data are mean of fold induction relative to DMSO ± SEM of four independent experiments; values significantly different from DMSO: *p < 0.05 (one-way ANOVA with Dunnett’s test). In (**L**), putative molecular mechanisms for overcoming GEM resistance by BBIT20. (1) Disruption of the BRCA1-BARD1 interaction, leading to BRCA1 nuclear-cytoplasmic shuttling and degradation, resulting in increased DNA damage, genomic instability, and promotion of cell death; (2) Inhibition of the multidrug resistance P-glycoprotein activity, reducing GEM efflux, and enhancing intracellular GEM accumulation; (3) Upregulation of human equilibrative nucleoside transporters (hENT1), increasing GEM uptake; (4) Downregulation of GEM metabolism-related resistance enzymes, including ribonucleotide reductase 1 and 2 (RRM1/2) and thymidylate synthase (TS), which decreases the alternative nucleotide pool. RRM1 and RRM2 convert ribonucleotide diphosphates (CDP) to deoxyribonucleotide diphosphates (dCDP), that are further converted into deoxycytidine triphosphate (dCTP), the competitive inhibitor of GEM. TS increases the alternative nucleotide pool by converting deoxyuridylate (dUMP) to deoxythymidylate (dTMP)

Given the high propensity of PDAC to develop therapeutic resistance, we analysed the anticancer activity of BBIT20 in a GEM-resistant PDAC cell line. Conversely to GEM, which exhibited a significantly higher IC_50_ value in GEM-resistant (9.82 ± 0.03 µM) compared to parental (0.70 ± 0.09 µM) MIA-PaCa-2 cells (Fig. 5A), BBIT20 demonstrated comparable IC_50_ values in both GEM-resistant (5.52 ± 0.03 µM) and parental (6.18 ± 0.04 µM) MIA-PaCa-2 cells (Fig. 5B). These findings showed that GEM-resistant PDAC cells did not exhibit cross-resistance to BBIT20. In fact, in these cells, BBIT20 significantly enhanced apoptosis (annexin V-positive cells) after 48 h of treatment with 3 and 6 µM (Fig. 5C).

As reported [53], we confirmed the increased expression of ATP Binding Cassette (ABC) Subfamily B Member 1 (ABCB1)/P-glycoprotein (P-gp) in GEM-resistant MIA-PaCa-2 cells (Supplementary material, Figure S4A and B). Although BBIT20 did not alter P-gp protein expression levels (Supplementary material, Figure S4C and D), 6 µM of BBIT20 significantly inhibited P-gp activity in GEM-resistant MIA-PaCa-2 cells (Fig. 5D and E), as indicated by increased intracellular accumulation of the fluorogenic P-gp substrate hydrolysis product (Fig. 5D). Notably, the inhibitory effect of BBIT20 on P-gp proved to be superior to that of verapamil (positive control), for a 17-fold lower concentration (Fig. 5D and E).

We also evaluated the impact of BBIT20 on the migration and invasion of GEM-resistant MIA-PaCa-2 cells. The antimigratory activity of BBIT20 on these cells was assessed by the wound-healing assay. The results showed that 1.5 µM of BBIT20 (IC_10_, with no significant effect on cell proliferation) significantly inhibited GEM-resistant MIA-PaCa-2 cell migration after 24, 30 and 48 h of treatment (Fig. 5F and G). Additionally, in the chemotaxis cell migration assay, 24 h of treatment with 1.5 µM of BBIT20 significantly impaired the ability of GEM-resistant MIA-PaCa-2 cells to migrate through a microporous membrane and invade through an extracellular matrix layer (Fig. 5H). Accordingly, BBIT20 inhibited EMT markers in GEM-resistant MIA-PaCa-2 cells. In fact, 48 h treatment with 1.5 and 6 µM of BBIT20 increased E-cadherin expression and decreased the protein levels of β-catenin, ZEB1 and matrix metalloproteinase-9 (MMP-9) (Fig. 5I).

In agreement with prior studies [54], we confirmed that GEM-resistant MIA-PaCa-2 cells exhibited elevated protein levels of RRM2 and decreased levels of equilibrative nucleoside transporter 1 (ENT1) (supplementary material, Figure S4A and B). RRM2, an enzyme involved in DNA synthesis and repair by catalysing the reduction of ribonucleotides, counteracts GEM’s mechanism of action by maintaining GEM-competitive deoxyribonucleotides levels [55]. Its upregulation has been linked to GEM resistance, making RRM2 silencing or downregulation a promising strategy against this resistance [47]. Accordingly, 3 and 6 µM of BBIT20 reduced RRM2 expression levels, in GEM-resistant MIA-PaCa-2 (Fig. 5J). On the other hand, ENT1, which facilitates the import of nucleosides like GEM into the cytosol [55], exhibited increased protein levels after BBIT20 treatment (Fig. 5J). These data further supported the potential of BBIT20 to sensitize GEM-resistant MIA-PaCa-2 cells to GEM.

As observed in PANC-1 (Fig. 1K) and MIA-PaCa-2 cells (Fig. 1L), 6 µM of BBIT20 significantly decreased the expression of miR-20a, in GEM-resistant MIA-PaCa-2 cells, after 24 h of treatment (Fig. 5K). In addition, miR-20a was previously associated with EMT regulation, in PDAC cells [56], supporting the dual effect of BBIT20 in reverting EMT processes and overcoming drug resistance (Fig. 5L). Of note, although downregulation of miR-200c has been linked to chemoresistance, including in GEM-resistant cells [57], BBIT20 did not significantly affect miR-200c levels, in GEM-resistant MIA-PaCa-2 cells (data not shown).

Altogether, these results emphasized the great potential of BBIT20 in addressing GEM-resistant PDAC cells by promoting cell death, suppressing cell motility, and modulating critical molecular targets associated with EMT and GEM resistance.

### BBIT20 sensitizes WtBRCA PDAC cells to OLAP and GEM-resistant PDAC cells to GEM

**Fig. 6:**
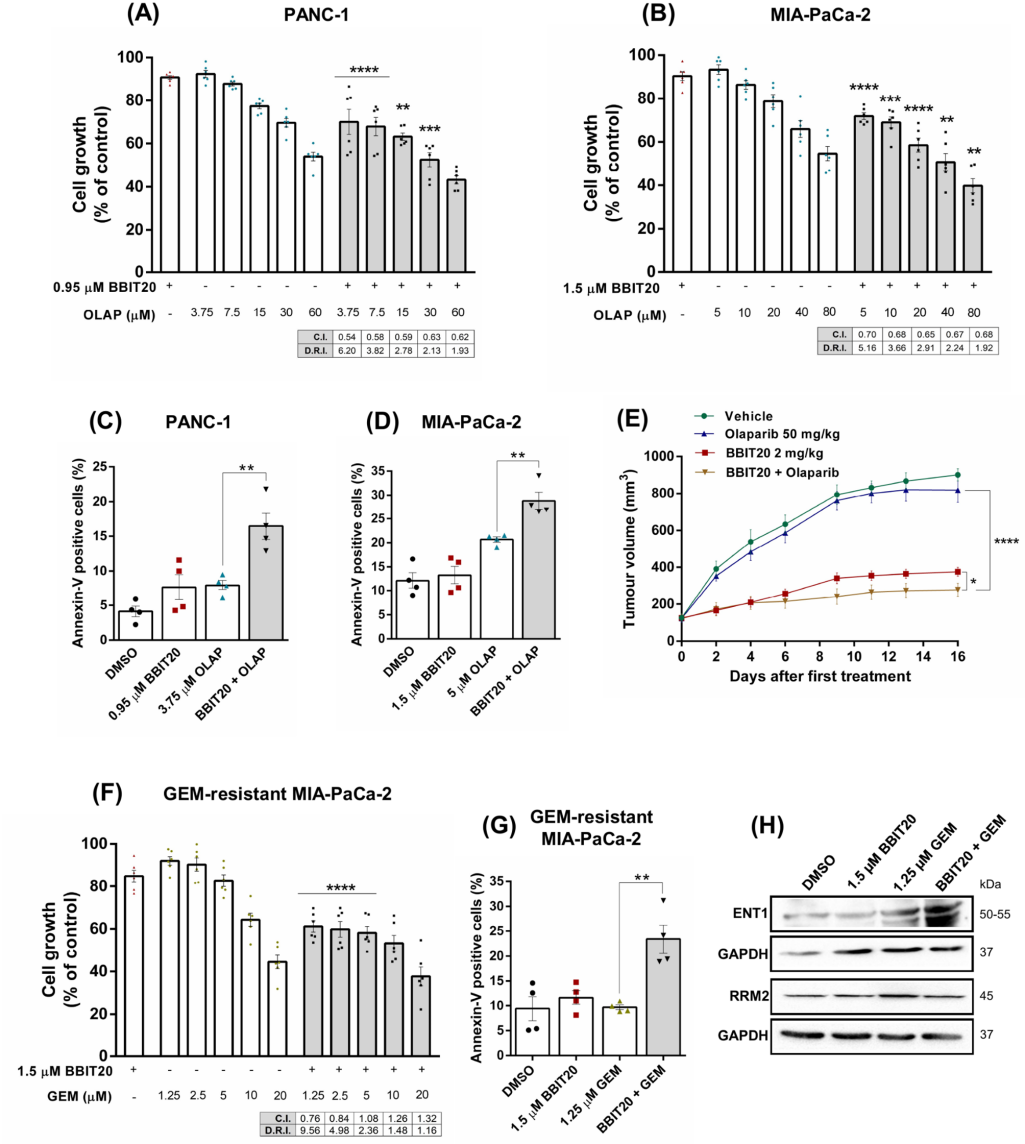
BBIT20 displays synergistic effects in combination with OLAP and GEM, enhancing their apoptotic potential, in PDAC cells, and sensitizing GEM-resistant PDAC cells to GEM. In (**A**, **B**), effects of the IC_10_ value of BBIT20, both alone and in combination with a concentration range of OLAP, on the proliferation of (**A**) PANC-1 and (B) MIA-PaCa-2 cells, were determined by SRB assay after 48 h of treatment. In (**C**, **D**), effect of BBIT20, both alone and in combination with OLAP, on apoptosis induction, in (**C)** PANC-1 and (**D**) MIA-PaCa-2, after 48 h of treatment; data are mean ± SEM of four independent experiments; values significantly different from OLAP: **p < 0.01 (one-way ANOVA with Dunnett’s test). In (**E**), tumour growth over time (days after the first treatment) in heterotopic xenograft mouse models of PDAC. NSG mice with subcutaneously implanted PANC-1 cells were treated with vehicle (5% DMSO in corn oil), olaparib (50 mg/kg), BBIT20 (2 mg/kg) or the combination of BBIT20 with olaparib. Treatments were administered via intraperitoneal injection three times a week (seven administrations in total). Tumour growth is presented as mean ± SEM of eight animals per group. Tumour growth for the drug combination significantly different from BBIT20 or olaparib alone: *p < 0.05, ****p < 0.0001 (two-way ANOVA with Tukey’s test). In (**F**), effects of the IC_10_ concentration of BBIT20, both alone and in combination with a concentration range of GEM, on proliferation of GEM-resistant MIA-PaCa-2 cancer cells, were determined by SRB assay after 48 h of treatment. In (**A**, **B**, **F**), growth obtained with DMSO was set as 100%; data are mean ± SEM of six independent experiments (two replicates each); growth significantly different from OLAP or GEM alone: **p < 0.01, ***p < 0.001, ****p < 0.0001 (two-way ANOVA with Dunnett’s test). C.I. and D.R.I. values were obtained using CompuSyn software (C.I. < 1, synergy; 1 < C.I. < 1.1, additive effect; C.I. > 1.1, antagonism). In (**G**), effect of BBIT20, both alone and in combination with GEM, on apoptosis induction in GEM-resistant MIA-PaCa-2 cells, after 48 h of treatment; data are mean ± SEM of four independent experiments; values significantly different from GEM: **p < 0.01 (one-way ANOVA with Dunnett’s test). In (**H**), effect of 48 h of treatment with 1.5 µM of BBIT20, 1.25 µM of GEM and the combination of BBIT20 and GEM, on ENT1 and RRM2 protein levels, in GEM-resistant MIA-PaCa-2 cells; representative immunoblots of four independent experiments are shown; GAPDH was used as a loading control

Based on the induction of HR deficiency (BRCAness phenotype), we investigated the potential synergistic effects of combining BBIT20 with the PARPi OLAP, in wtBRCA PDAC cells. For that, BBIT20, at a concentration that showed no significant impact on cancer cell growth (IC_10_), was tested in combination with a concentration range of OLAP. The results revealed that BBIT20 significantly enhanced the growth inhibitory activity of OLAP compared to OLAP alone, in PANC-1 and MIA-PaCa-2 cells (Fig. 6A and B). The combination index (C.I.) and dose reduction index (D.R.I.) values were determined by multiple drug-effect analysis for each combination, showing synergistic effects between BBIT20 and OLAP across all tested combinations (C.I. < 1), and a substantial reduction of the effective dose of OLAP (Fig. 6A and B). Particularly, in PANC-1 cells, the combination of 0.95 µM of BBIT20 with 3.75 µM of OLAP resulted in a 6.20-fold reduction of the effective dose of OLAP (Fig. 6A); in MIA-PaCa-2 cells, the combination of 1.5 µM of BBIT20 with 5 µM of OLAP caused a 5.16-fold dose reduction of OLAP (Fig. 6B). Consistently, in these combinations, BBIT20 significantly increased the apoptotic potential of OLAP, in both PANC-1 (Fig. 6C) and MIA-PaCa-2 (Fig. 6D) cells.

In a heterotopic xenograft mouse model of PANC-1 cells, BBIT20 demonstrated potent antitumour activity (Fig. 6E). In contrast, as anticipated in a wtBRCA context, OLAP did not significantly inhibit tumour growth (Fig. 6E). Importantly, consistent with our findings in 2D and 3D PDAC models, we observed a significant enhancement of the antitumour activity of OLAP in combination with BBIT20 (Fig. 6E).

Additionally, to evaluate the ability of BBIT20 to sensitize GEM-resistant MIA-PaCa-2 cells to GEM, a single dose of BBIT20 (IC_10_) was tested in combination with a concentration range of GEM. The results revealed an enhancement of the growth inhibitory activity of GEM by BBIT20 compared to GEM alone (Fig. 6F). As with OLAP, the combination and dose reduction indexes were determined. For the lowest concentrations of GEM, synergistic effects (C.I. < 1) were observed associated with a marked reduction of the effective dose (D.R.I.) of GEM (Fig. 6F). In particular, a synergistic effect was achieved with 1.5 µM of BBIT20 and 1.25 µM of GEM, resulting in a 9.56-fold dose reduction of GEM. Consistently, BBIT20 also significantly increased the apoptotic potential of GEM, in GEM-resistant cells (Fig. 6G).

Notably, in line with experiments using BBIT20 alone (Fig. 5J), in combination with GEM, BBIT20 decreased RRM2 and increased ENT1 protein levels (Fig. 6H), which further supported the potential of the compound to sensitize GEM-resistant PDAC cells to GEM.

Collectively, these results underscored the considerable potential of BBIT20 in combination with standard-of-care therapies, such as OLAP and GEM, promoting their cytotoxicity while reducing effective doses and associated side effects.

### BBIT20 inhibits the growth of patient-derived PDAC organoids, enhancing their sensitivity to OLAP and GEM

**Fig. 7:**
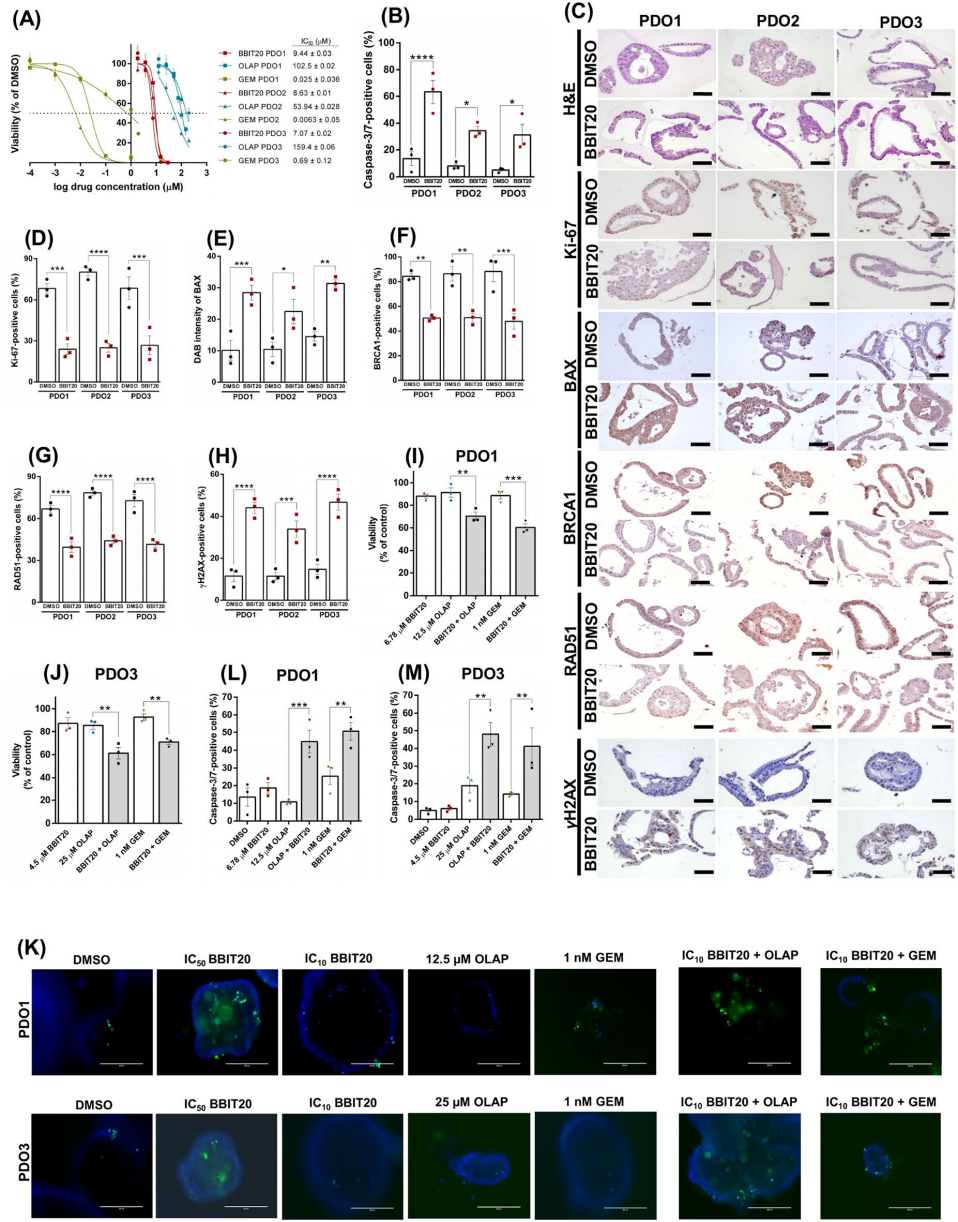
BBIT20 inhibits the growth of patient-derived organoids of PDAC, enhancing the cytotoxic effects of OLAP and GEM by inducing apoptosis. In (**A**), dose-response curves of BBIT20, OLAP and GEM in wtBRCA PDOs, determined by CellTiter-Glo^®^ assay, after 72 h of treatment. Data are mean ± SEM of three independent experiments. In (**B**), quantification of activated caspase-3/7-positive cells in organoid culture treated for 72 h with vehicle (DMSO) or IC_50_ of BBIT20 (9.44 µM for PDO1, 8.63 µM for PDO2, and 7.07 µM for PDO3). Data are mean ± SEM of three independent experiments; values significantly different from DMSO: *p < 0.05, ****p < 0.0001 (two-way ANOVA with Sidak’s test). In (**C**), haematoxylin and eosin (H&E) and immunohistochemistry staining (Ki-67, BAX, BRCA1, RAD51, and γH2AX) of organoid cultures treated for 72 h with vehicle (DMSO) or IC_50_ of BBIT20 (scale bar = 50 µm; 400× magnification). In (**D**-**H**), quantification of (D) Ki-67, (**E**) BAX staining by 3,3’-diaminobenzidine (DAB) intensity, (**F**) BRCA1, (**G**) RAD51, and (**H**) γH2AX, in PDOs. Data are mean ± SEM of three independent experiments; values significantly different from DMSO: *p < 0.05, **p < 0.01, ***p < 0.001, ****p < 0.0001 (one-way ANOVA with Sidak’s test). In (**I**, **J**), viability effects in PDO1 (**I**) and PDO3 (J) treated with the IC_10_ concentration of BBIT20 (6.78 µM for PDO1 and 4.5 µM for PDO3), OLAP or GEM alone, and BBIT20 (IC_10_) plus OLAP or GEM, after 72 h of treatment. Drug combination studies were performed using CellTiter-Glo^®^. Viability obtained with control (DMSO) was set as 100%; data are mean ± SEM of three independent experiments. Organoids viability significantly different from OLAP or GEM alone: **p < 0.01, ***p < 0.001 (one-way ANOVA with Sidak’s test). In (**K**), activated caspase-3/7 staining (CellEvent™ Caspase-3/7 green detection), in PDO1 and PDO3 treated with vehicle (DMSO), IC_10_ and IC_50_ of BBIT20, OLAP or GEM alone, and BBIT20 (IC_10_) plus OLAP or GEM, after 72 h of treatment. Representative images of caspase-3/7 staining (green), and nuclear Hoechst staining (blue), are shown; magnification = 400×, scale bar = 200 μm. In (**L**, **M**), quantification of activated caspase-3/7-positive cells in (**L**) PDO1 and (**M**) PDO3 treated with vehicle (DMSO), IC_10_ of BBIT20, OLAP or GEM alone, after 72 h of treatment. Data are mean ± SEM of three independent experiments, values significantly different from OLAP or GEM: **p < 0.01, ***p < 0.001 (one-way ANOVA with Sidak’s test)

PDAC organoids represent a robust 3D preclinical model for predicting drug sensitivities, effectively recapitulating the physiology and genetic diversity of PDAC. Studies have demonstrated that the treatment responses of organoids to standard chemotherapeutics closely reflect those of the patients from whom they were derived, supporting the potential of PDAC organoids to advance personalized drug screening studies [58]. Based on this, the cytotoxic effect of BBIT20 was evaluated in 3D primary PDAC patient-derived organoids (PDOs), by CellTiter-Glo^®^ assay. BBIT20 was tested in three PDOs harbouring wtBRCA (characterization in supplementary material, Table S3). The IC_50_ values of BBIT20 ranged from 7.07 ± 0.02 µM to 9.44 ± 0.03 µM (Fig. 7A). Consistently with our previous results (Fig. 1B and C), the growth inhibitory effect of BBIT20 was much superior to that of OLAP in all wtBRCA PDOs of PDAC (Fig. 7A). Notably, the sustained cytotoxic effect of 7.07 ± 0.02 µM in the most resistant PDO3, which demonstrated resistance to both OLAP and GEM, further supported the efficacy of BBIT20 against drug resistant PDAC. The apoptotic potential of BBIT20 was thereafter assessed by checking the activated caspase-3/7 cells (Fig. 7B and K). Treatment of PDOs with BBIT20 at respective IC_50_ values (9.44 µM for PDO1, 8.63 µM for PDO2, and 7.07 µM for PDO3), for 72 h, resulted in a significant increase in the percentage of caspase-3/7-positive cells, especially in PDO1 (Fig. 7B and K). The haematoxylin and eosin (H&E) staining showed that BBIT20 did not induce any morphological changes in PDOs of PDAC, as demonstrated by the preservation of PDAC glandular morphology (Fig. 7C). The inhibition of PDOs proliferation by BBIT20 was further evidenced by the significant reduction of Ki-67-positive staining (Fig. 7C and D). Moreover, the significant increase of BAX staining corroborated an enhancement of cell death in response to BBIT20 treatment (Fig. 7C and E). The inhibitory effect of BBIT20 on HR DNA repair was also confirmed in PDOs of PDAC, as denoted by the reduction of BRCA1- (Fig. 7C and F) and RAD51- (Fig. 7C and G) positive cells. Additionally, the genotoxic effect of BBIT20 was supported by the increase of γH2AX-positive cells in these PDOs (Fig. 7C and H).

Combinatory regimens of BBIT20 with OLAP or GEM were also evaluated in PDO1 and PDO3, using CellTiter-Glo^®^, after 72 h of treatment. In accordance with our previous results (Fig. 6), BBIT20 sensitized PDO1 (Fig. 7I) and PDO3 (the most resistant PDO; Fig. 7J) to OLAP and GEM, enhancing their growth inhibitory effects. The apoptotic potential of BBIT20 in combination with OLAP or GEM was further evaluated by assessing the levels of activated caspase-3/7 cells (Fig. 7K-M). Consistently, the combination of 6.78 µM of BBIT20 with 12.5 µM of OLAP or 1 nM of GEM (in PDO1), and 4.5 µM of BBIT20 with 25 µM of OLAP or 1 nM of GEM (in PDO3), significantly enhanced apoptosis compared to chemotherapeutics alone (Fig. 7K-M).

Altogether, the results in patient-derived organoids emphasized the potent growth inhibitory effect of BBIT20 on wtBRCA PDAC and its potential to sensitize these cells to standard-of-care therapies, including OLAP and GEM.

### BBIT20 reduces tumour growth, improves overall survival, and inhibits liver metastasis formation in an orthotopic xenograft mouse model of PDAC

To further validate the anticancer effect of BBIT20 against PDAC, PANC-1 cells were orthotopically implanted in the pancreas of Rag2^−/−^ IL2rg^−/−^ mice, generating PDAC tumours. Following confirmation of tumour growth, mice were treated with either vehicle or 2 mg/kg of BBIT20, three times a week, until natural death or euthanasia due to severe symptoms.

**Fig. 8:**
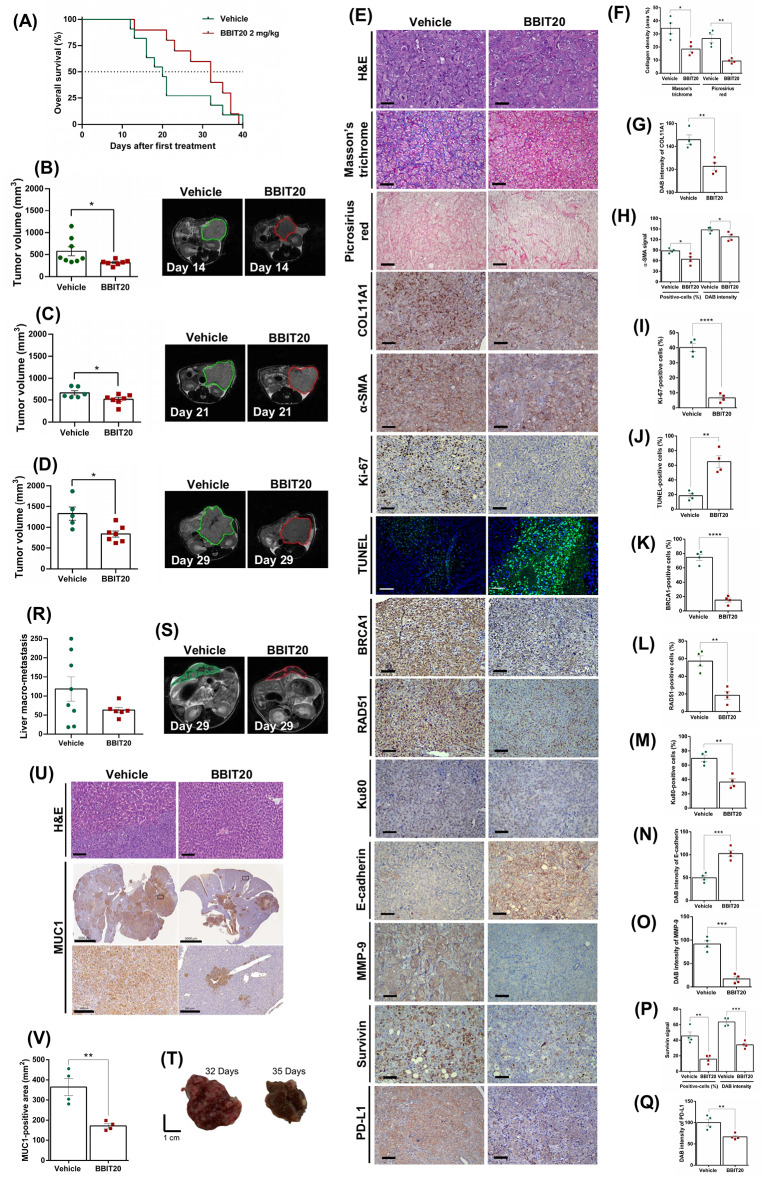
BBIT20 improves overall survival, inhibits tumour growth and reduces liver metastasis in an orthotopic xenograft mouse model of PDAC. Rag2^−/−^ IL2rg^−/−^ mice orthotopically inoculated into the pancreas with PANC-1 cells and treated (intraperitoneal injection) three times per week with vehicle (5% DMSO in corn oil) or BBIT20 (2 mg/kg). In (**A**), Kaplan–Meier survival curves of mice over time (days after the first treatment). Survival of BBIT20-treated mice compared to vehicle: *p* > 0.05 (log-rank (Mantel–Cox) test). In (B-D), effect of BBIT20 on tumour growth, evaluated by magnetic resonance imaging (MRI), after (**B**) 14, (**C**) 21, and (**D**) 29 days of the first treatment. Representative images of tumour volume shown for the three timepoints using the same animals of each experimental group over time. Tumour volume represented as mean ± SEM. Tumour growth of BBIT20-treated group significantly different from vehicle: *p < 0.05 (unpaired *t*-test). In (**E**), representative images of H&E, Masson’s trichrome, Picrosirius red, COL11A1, α-SMA, Ki-67, TUNEL, BRCA1, RAD51, Ku80, E-cadherin, MMP-9, survivin and PD-L1 staining (scale bar = 50 μm, 200× magnification) of vehicle and BBIT20-treated PDAC after 32 and 35 days of treatment, respectively. In (**F**-**Q**), quantification of (**F**) percentage area of tumour collagen density based on Masson’s trichrome and Picrosirius staining, (**G**) COL11A1 signal intensity, (**H**) α-SMA-positive cells and signal intensity, (**I**) Ki-67-positive cells, (**J**) TUNEL-positive cells, (**K**) BRCA1-positive cells, (**L**) RAD51-positive cells, (**M)** Ku80-positive cells, (**N**) E-cadherin signal intensity, (**O**) MMP-9 signal intensity, (**P**) survivin-positive cells and signal intensity, and (**Q**) PD-L1 signal intensity, of four vehicle- and BBIT20-treated tumours. Values significantly different from vehicle: *p < 0.05, **p < 0.01, ***p < 0.001, ****p < 0.0001 (unpaired *t*-test). In (**R**-**T**), effect of BBIT20 on liver macro-metastases. In (**R**), quantification of liver macro-metastasis on mice endpoint, represented as mean ± SEM, values not significantly different from vehicle: *p* > 0.05 (unpaired *t*-test). In (**S**-**T**), representative images of livers and macro-metastases of the same animals of each experimental group. In (**S**), livers with macro-metastasis observed by MRI on day 29 after the first treatment, and in (**T**) corresponding livers collected on mice endpoint (after 32 and 35 days of first treatment). In (**U**), representative H&E (scale bar = 50 μm, 200× magnification) and MUC1 immunohistochemical staining of vehicle and BBIT20-treated livers (upper images: scale bar = 5000 μm, 2.3× magnification; lower images: scale bar = 300 μm, 40× magnification), treated for 32 and 35 days, respectively, highlighting liver metastatic foci. In (**V**), quantification of liver MUC1-positive areas, underscoring the area of micro-metastasis of four vehicle- and BBIT20-treated livers. MUC1-positive area significantly different from vehicle: **p < 0.01 (unpaired *t*-test)

BBIT20 increased the median overall survival of mice from 20 days (vehicle) to 32 days (Fig. 8A). Although the log-rank (Mantel-Cox) test did not reveal a statistically significant difference between the survival curves, the hazard ratio analysis indicated a potential benefit from BBIT20 treatment. In fact, the hazard ratio for BBIT20-treated group of 0.6395 was lower than that of the control group of 1.564, denoting a reduced risk of death with BBIT20 treatment. Importantly, no morbidity signs nor significant variations of body weight (Figure S5A) were observed in BBIT20-treated mice compared to vehicle. Histological assessment by H&E staining showed no significant morphological differences in major organs (liver, lungs, kidneys, and spleen) between the vehicle- and BBIT20-treated mice (Figure S5B). This is in line with our previous study [24], in which haematological and biochemical analysis revealed no signs of toxicity in mice, at the same dose of BBIT20 (2 mg/kg) administered in the current work. Collectively, these data demonstrated that BBIT20 has a favourable safety profile at its effective dose in vivo.

During the experiment, tumour growth was monitored by magnetic resonance imaging (MRI), showing that BBIT20 significantly inhibited PDAC growth, namely at 14, 21 and 29 days after the first treatment (Fig. 8B-D). Histological examination by H&E staining showed no significant differences between tumours from vehicle- and BBIT20-treated mice (Fig. 8E). However, an evident reduction in total fibrillar collagen content was noted in BBIT20-treated tumours, as demonstrated by the diminished levels of blue- and red-stained collagen in Masson’s trichrome and Picrosirius red staining, respectively (Fig. 8E and F). These findings suggested that BBIT20 effectively reduced extracellular matrix fibrotic tissue, thereby mitigating tumour desmoplasia. Accordingly, a significant reduction in collagen type XI alpha 1 (COL11A1) levels could also be observed in BBIT20-treated tumours (Fig. 8E and G), a collagen subtype typically overexpressed in PDAC that is associated with tumour progression, metastasis and poor prognosis [59]. In line with these results, we evaluated alpha smooth muscle actin (α-SMA), a marker of activated fibroblasts and myofibroblasts, which plays a key role in fibrosis and tumour microenvironment. Consistently, BBIT20 treatment significantly reduced α-SMA-positive cells in PDAC tissues (Fig. 8E and H), further supporting its antifibrotic effect.

Moreover, BBIT20-treated tumours exhibited decreased Ki-67-positive cells (Fig. 8E and I), indicating an antiproliferative activity. Furthermore, an increased number of Terminal deoxynucleotidyl transferase dUTP Nick End Labelling) (TUNEL)-positive cells (Fig. 8E and J), indicating higher levels of DNA fragmentation, was observed in BBIT20-treated tumours compared to vehicle. In addition, BBIT20-treated tumours showed a reduced number of BRCA1- (Fig. 8E and K) and RAD51- (Fig. 8E and L) positive cells, supporting the inhibitory effect of BBIT20 on HR. Also, in alignment with the NHEJ reporter assay in PANC-1 cells (Fig. 4H), we confirmed in vivo a reduction in Ku80 levels (Fig. 8E and M), which is a core component of the NHEJ pathway [60].

To further confirm the effect of BBIT20 on EMT and invasion, an increase of E-cadherin (Fig. 8E and N) and a decrease of MMP-9 (Fig. 8E and O) staining intensities were observed in BBIT20-treated tumours, supporting the inhibitory effect of BBIT20 on tumour dissemination. We further evaluated the expression of survivin, a key regulator of the cell cycle progression and apoptosis inhibition, that is frequently overexpressed in PDAC and associated with poor prognosis, chemoresistance, tumour proliferation and metastatic potential [43]. Accordingly, survivin was found decreased in BBIT20-treated tumours (Fig. 8E and P), which is in line with an induction of cell death by BBIT20, counteracting tumour growth and dissemination, and improving PDAC prognosis. Consistent with the tumour microenvironment modulation and EMT inhibition induced by BBIT20, we assessed programmed death-ligand 1 (PD-L1), a poor prognostic marker that mediates immune evasion in tumour cells, within vehicle- and BBIT20-treated PDAC tumours. Notably, BBIT20 treatment resulted in a reduction in PD-L1 staining intensity (Fig. 8E and Q), suggesting a potential reactivation of the tumour immune response to recognize and destroy tumour cells, thereby restoring effective antitumor immune responses [61].

Additionally, a potential reduction in the number of liver macro-metastasis was observed in BBIT20-treated group, particularly noted by MRI during the experiment (Fig. 8S) and in liver specimens collected at the mice endpoints (Fig. 8R and T). Notably, measurements of volume (Supplementary material, Figure S5C) and weight (Supplementary material, Figure S5D) further indicated a potential reduction in these liver parameters for the BBIT20-treated group. In fact, histopathological analysis revealed that BBIT20 reduced the metastatic burden within the liver (Fig. 8U). Mucin 1 (MUC1) staining was used to identify tumour cells in liver tissues, confirming a reduction of the liver metastatic foci area in BBIT20-treated mice compared to the vehicle group (Fig. 8U and V).

Altogether, the collective outcomes of reduced tumour growth, enhanced overall survival, and significant antimetastatic effects emphasised the promising therapeutic potential of BBIT20 for treating PDAC patients.

## Discussion

Among the DNA repair inhibitors, the PARPi OLAP remains a leading targeted therapy for PDAC patients with mutBRCA, but it has limited efficacy in wtBRCA (HR-proficient) cases [1]. Herein, we unveil the anticancer therapeutic potential of BBIT20 as an innovative BRCAness inducer that effectively inhibits HR DNA repair, in PDAC regardless of BRCA status. In fact, as previously observed in ovarian and breast cancer cells [24], BBIT20 showed a significant antiproliferative effect on PDAC cells carrying either wt or mutBRCA. In contrast, OLAP exhibited a reduced growth inhibitory effect on wtBRCA PDAC cells, as expected. Given the low prevalence of germline BRCA mutations in PDAC (over 5–10%) [14], which implies a large percentage of HR-proficient PDAC patients not covered by OLAP and more resistant to DNA-damaging standard therapies, we aimed to further investigate the effectiveness of BBIT20, either alone or in combination therapy, in wtBRCA PDAC.

Consistently, in patient-derived organoids (PDOs) of wtBRCA PDAC, with distinct drug sensitivities, BBIT20 showed a potent growth inhibitory activity, markedly surpassing that of OLAP. In these PDOs and wtBRCA PDAC cells, the BBIT20-induced growth inhibition was associated with cell cycle arrest, apoptosis induction, and genotoxicity. A marked HR deficiency, with significant downregulation of key players in HR DNA damage repair, such as BRCA1, BRCA2, RAD50, RAD51, RAD52 and RAD54, was also observed.

The BRCA1-BARD1 heterodimer plays a central role in HR pathway [1, 62]. Inhibition of this interaction results in a deficient HR DNA repair due to BRCA1 shuttling to the cytoplasm with its proteasomal degradation, and the destabilization of other HR-related proteins connected to this protein-protein complex, such as BRCA2 and RAD51 [1, 62]. This process promotes genomic instability and cell death, making it a promising anticancer strategy, particularly for wtBRCA tumours, by enhancing their susceptibility to DNA-damaging agents [1, 63]. In fact, given the high prevalence of mutp53 in PDAC (50–75% of PDAC cases) [64], which is associated with resistance of cancer cells to chemotherapeutic agents by directly compromising BRCA1 shuttling to cytoplasm [65], BBIT20 represents a promising strategy to overcome cancer drug resistance in mutp53 PDAC, by inducing BRCA1 nuclear export (usually mediated by a functional p53) [65]. In our early study, we provided evidence that BBIT20 disrupted the BRCA1-BARD1 interaction in ovarian and breast cancer cells [24]. In the present work, we further confirmed the disruption of this heterodimer, with subsequent BRCA1 nucleocytoplasmic translocation and protein degradation, in wtBRCA and mutp53 PDAC. Importantly, a yeast two-hybrid assay, reproducing this protein-protein interaction, could validate a selective inhibitory effect of BBIT20 on BRCA1-BARD1 complex. Overall, these data support BBIT20 as the first-in-class inhibitor of HR by disrupting the BRCA1-BARD1 interaction.

Some other protein-protein interaction disruptors have been disclosed as HR inhibitors. Namely, a family of triazole derivatives, CAM833, and dihydroquinolone pyrazoline derivatives were reported as inhibitors of the BRCA2-RAD51 interaction [66–68]. Like BBIT20, these compounds can induce BRCAness, affecting RAD51 recruitment at DNA double-strand breaks [66–68]. However, they show a more restricted effect on the final execution of HR DNA repair [69, 70], along with physicochemical and pharmacokinetic limitations [68], which ultimately prevented their progression to clinical trials.

While a functional BRCA1-BARD1 heterodimer is essential for executing HR DNA repair in non-malignant cells, we previously demonstrated a low cytotoxicity of BBIT20 on these cells, associated with reduced side effects in mice [24]. This was further corroborated in our current study, as we observed no apparent undesirable toxicity in mice following prolonged administration of the compound. This can be attributed to the compensation of HR inhibition by other functional DNA repair mechanisms [71], enabling non-malignant cells to evade the cytotoxic effects of BBIT20.

BBIT20 can sensitize wtBRCA PDAC cells to DNA-damaging agents, like OLAP and GEM, primarily through the induction of synthetic lethality via the mutual inhibition of different DNA repair pathways [1]. In fact, it showed significant synergistic effects with both OLAP and GEM, greatly enhancing their cytotoxicity, in wtBRCA PDAC cells. Importantly, similar synergistic effects were also observed with GEM, in GEM-resistant PDAC cells. Additionally, a significant enhancement of OLAP and GEM cytotoxicity by BBIT20 was achieved in PDOs of wtBRCA PDAC. Notably, in vivo studies revealed that BBIT20 markedly improved the antitumour activity of OLAP in PDAC tumours harbouring wtBRCA. These synergistic combinations represent a notable clinical advancement in the treatment of PDAC. They may extend the application of standard-of-care drugs, particularly OLAP, to HR-proficient PDAC patients, who typically do not respond or have a poor response to these therapies. Moreover, this approach may allow for a substantial reduction in the effective doses of OLAP and GEM, mitigating their toxicity and providing patients with better-tolerated treatment regimens, ultimately enhancing their quality of life.

As previously noted, one contributing factor to the poor prognosis of PDAC is its high propensity for developing therapeutic resistance [1, 55]. Acquired resistance to GEM, the cornerstone of PDAC therapy, remains a clinical concern [55]. Based on this, the anticancer activity of BBIT20 was further evaluated in GEM-resistant PDAC cells. Although cancer cells that develop resistance to a particular drug often undergo molecular changes that may also confer resistance to additional therapies [72], GEM-resistant PDAC cells did not exhibit cross-resistance to BBIT20. In fact, BBIT20 demonstrated similar antiproliferative effects on parental and GEM-resistant PDAC cells. It is also noteworthy that in these GEM-resistant PDAC cells, distinct molecular mechanisms of reversion of GEM resistance by BBIT20 were evidenced (Fig. 5L). Particularly, BBIT20 was found to upregulate the hENT1 transporter, which enhances GEM uptake. In fact, decreased hENT1 levels are commonly observed in GEM-resistant PDAC cells [54]. In accordance with this, higher levels of hENT1 expression have been associated with improved overall survival in PDAC patients treated with GEM [55, 73]. BBIT20 also downregulated key enzymes involved in pyrimidine metabolism, such as RRM1, RRM2 and TS (Fig. 5L), which are linked to the development of GEM resistance. In fact, this leads to a depletion of the deoxyribonucleotide pool of competitive inhibitors of GEM, required for DNA synthesis and repair [55, 74].

 Accordingly, both hENT1 and RRM1/2 are GEM-related biomarkers and have prognostic value in specific treatment setting in PDAC [75, 76]. As such, the regulation of hENT1 and RRM1/2 expression levels by BBIT20 in GEM-resistant cells underscored its potential to improve patients’ response to GEM and subsequent survival outcomes. Interestingly, BBIT20 further regulated the expression of miRNAs implicated in PDAC prognosis, particularly reducing miR-20a levels (including in GEM-resistant cells), which is correlated with EMT regulation [56], and GEM chemosensitivity [47]. Of note, a more stable form of GEM, 4-(N)-Stearoyl GEM or S-GEM was synthesized to improve the anticancer activity of GEM in PDAC, showing promise in overcoming GEM resistance by increasing hENT1 expression, while reducing RRM1 levels [77]. Notably, BBIT20 offers a therapeutic advantage over S-GEM by not only modulating key regulators of GEM metabolism, but also by inhibiting DNA damage repair mechanisms, providing a more robust therapeutic strategy.

P-gp is a transporter often implicated in multidrug resistance due to its role in promoting the efflux of drugs such as GEM [78]. In line with this, our data indicated that while BBIT20 did not alter the protein expression levels of P-gp, it effectively inhibited its drug efflux functionality in GEM-resistant PDAC cells. Indeed, strategies focused on inhibiting P-gp-mediated drug efflux have shown significant promise in reversing multidrug resistance phenotypes, thereby sensitizing cancer cells to standard chemotherapy [79]. One common P-gp inhibitor, the calcium-channel blocker verapamil, increases the retention of chemotherapeutics by competing for related binding sites on P-gp [80]. However, the clinical application of P-gp inhibitors, like verapamil, has been hindered by issues related to high toxicity, low potency, lack of specificity, substrate promiscuity, and unclear mechanisms of transport [81]. Notably, the inhibitory effect of BBIT20 on P-gp activity revealed to be much superior to that obtained with verapamil, in GEM-resistant PDAC cells. This further supported the great potential of BBIT20 to counteract drug resistance by effectively inhibiting P-gp activity. In fact, although cancer cells often develop resistance to DNA repair inhibitors [1], BBIT20 has not induced resistance in PDAC cells, potentially because of its robust inhibitory effect on P-gp activity. In fact, the proteomic analysis revealed that BBIT20 did not significantly affect key proteins associated with drug resistance in PDAC, specifically the ABC transporters ABCC4/multidrug resistance protein 4 (MRP4) and ABCC1/multidrug resistance protein 1 (MRP1), as well as cluster of differentiation 44 (CD44), SMAD family member 4 (SMAD4), fibronectin, epidermal growth factor receptor (EGFR), and signal transducer and activator of transcription 3 (STAT3). Furthermore, several histone deacetylases (HDACs) implicated in epigenetic regulation of drug resistance, including HDAC1, HDAC2, HDAC3, HDAC7, showed no significant expression changes. Notably, DNA methyltransferase 1 (DNMT1) was significantly downregulated (FC = 0.399; *p*-value = 2.88 × 10^− 3^), suggesting a potential shift towards DNA hypomethylation that may reverse aberrant methylation patterns, reactivating silenced tumour suppressor genes and enhancing the sensitivity of PDAC cells to the therapy. Furthermore, although BBIT20 effectively inhibits HR DNA repair, cancer cells commonly turn to the NHEJ pathway as a compensatory mechanism. Notably, BBIT20 also disrupts NHEJ, thereby preventing the emergence of this resistance mechanism. Overall, our findings underscored the significant potential of BBIT20 in addressing drug resistance, particularly to GEM, in PDAC. Such an ability is particularly relevant in drug-resistant settings, potentially improving therapeutic outcomes and overall survival rates of PDAC patients.

A substantial number of patients diagnosed with localized PDAC already exhibit metastases at the time of diagnosis [1, 82]. While GEM is commonly administered in metastatic PDAC, either alone or in combination with nab-paclitaxel, patients often exhibit considerable resistance to this treatment [5]. BBIT20 mitigated GEM-resistant PDAC cell migration and invasion by disrupting EMT processes, namely through upregulation of E-cadherin expression and downregulation of β-catenin, ZEB1, and MMP-9 levels. Furthermore, BBIT20 significantly increased the levels of miR-200c, which inhibits EMT by upregulating E-cadherin and suppressing the ZEB family [44–46], and downregulated the levels of miR-20a, also involved in EMT regulation [56]. Overall, these findings indicated that BBIT20 holds great promise in counteracting metastatic dissemination of PDAC, particularly in a setting of therapeutic resistance to GEM.

To validate in vivo the anticancer activity of BBIT20 in PDAC, an orthotopic xenograft mouse model was performed. In this assay, BBIT20 reduced tumour growth, which was associated with a marked inhibition of cell proliferation and enhancement of apoptotic cell death. Also in vivo, BBIT20 inhibited HR DNA repair as evidenced by the significant decrease of BRCA1 and RAD51 expression levels in tumour tissues. Survival curves in preclinical studies of PDAC often do not show substantial improvements due to the inherent aggressiveness of PDAC, reflecting the dismal 5-year survival rate lower than 8% [4]. Despite this, BBIT20 caused a visible improvement of the median overall survival of PDAC xenograft mice.

PDAC exhibits therapeutic limitations, since it is characterized by multiple barriers such as the dense and fibrotic stroma surrounding the tumour that acts as a barrier to drug delivery [83] and to the infiltration of effector immune cells, such as cytotoxic T lymphocytes [84]. Notably, BBIT20 reduced the collagen content, particularly COL11A1, and α-SMA in PDAC tumours. α-SMA is a protein commonly used as a marker of activated fibroblasts and myofibroblasts in tissues. In PDAC, these α-SMA-positive cells are often associated with pancreatic stellate cells (PSCs), which play a significant role in tumour microenvironment. When activated, PSCs contribute to the desmoplastic reaction commonly observed in PDAC. The activated PSCs can promote fibrosis, secrete extracellular matrix components, and release various growth factors and cytokines that can further support tumour growth and metastasis [85]. Collectively, these results demonstrate the significant impact of BBIT20 on extracellular matrix remodelling, not only reducing tumour growth and invasiveness, but also improving therapeutic response by effectively overcoming tumour desmoplasia.

Recently, RRM1 has been identified as a key inducer of extracellular matrix remodelling, enhancing mesenchymal characteristics and promoting the invasiveness of PDAC cells, in addition to its established role in regulating GEM resistance [86]. This evidence suggests a RRM1-mediated reduction on tumour fibrosis, particularly decreasing collagen density, and liver metastatic foci in BBIT20-treated PDAC. Furthermore, COL11A1, a marker of poor prognosis in PDAC, is a direct target of miR-20a, driving EMT and PDAC progression [56]. This further strengthens the observed correlation between the inhibition of tumour collagen content, EMT, and RRM1/2 protein levels by BBIT20. In addition, it is well-accepted that part of the failure of immunotherapy in PDAC is related to tumour-associated fibrosis [87]. By decreasing stromal density, BBIT20 could enhance drug penetration and improve drug efficacy, particularly potentiating immunotherapy response by facilitating immune cell access to the tumour. Accordingly, the reduction of α-SMA and PD-L1 expression, in tumour tissues, indicates that BBIT20 mitigates immunosuppression, fostering a tumour microenvironment more conducive to the activation and effectiveness of immune cells. By blocking PD-L1, its interaction with programmed cell death protein 1 (PD-1) on T cells is prevented, restoring T cell activity and allowing them to identify and destroy cancer cells. Collectively, these findings highlight the potential of BBIT20 as a promising candidate for combination therapy with immunotherapy in PDAC treatment [61].

Consistently, BBIT20 depleted EMT markers, particularly increasing E-cadherin and decreasing MMP-9 in tumour tissues, and markedly reduced the metastatic burden in the liver, a primary site of PDAC metastasis. In line with this, BBIT20 protected from enlarged liver. In fact, liver metastasis is a common complication in advanced PDAC, often leading to hepatomegaly due to tumour burden and liver inflammation [88]. Conversely to BBIT20, GEM has known limitations in preventing metastasis, since its primary function is to inhibit DNA synthesis in rapidly dividing PDAC cells [89]. Notably, a significant reduction of survivin was also found in BBIT20-treated tumour tissues. In fact, survivin is frequently overexpressed in PDAC and associated with chemoresistance, tumour proliferation and metastatic potential [43]. Its depletion further corroborated an improvement of PDAC prognosis by BBIT20.

## Conclusions

Limited treatment options and resistance to therapeutic interventions including chemotherapy, radiotherapy and immunotherapy, make PDAC one of the cancer types with poorest prognosis and survival rates [1, 82]. BBIT20 represents a first-in-class inhibitor of the BRCA1-BARD1 interaction, demonstrating significant promise as a monotherapy for PDAC by effectively reducing tumour growth and metastasis, while improving overall survival rates. It also showed great potential in combination with standard-of-care therapies, particularly OLAP and GEM, as it enhanced their cytotoxicity and allowed for a marked reduction of their effective doses against PDAC. These combinatory regimes could therefore translate into improved tolerability to standard-of-care therapies, reduced harmful side effects, better therapeutic response and clinical outcomes of PDAC patients. Notably, its potential as a stroma-targeting agent, capable of reducing fibrosis, associated with a pronounced depletion of PD-L1 expression, makes BBIT20 even more attractive for combination therapy, particularly with immunotherapy. Additionally, its ability to counteract drug resistance would represent an important clinical achievement in therapeutic development.

Overall, these preclinical findings underscore the great potential of BBIT20 as a novel multifaceted anticancer drug candidate for the treatment of PDAC, a challenging malignancy that still lacks effective therapeutic options.

## Electronic supplementary material

Below is the link to the electronic supplementary material.

Supplementary Material 1

## Data Availability

No datasets were generated or analysed during the current study.

## Abbreviations

C.I.: Combination index
CFUs: Colony-forming units
D.R.I.: Dose reduction index
ENT1: Equilibrative nucleoside transporter 1
GEM: Gemcitabine
HR: Homologous recombination
IC_50_: Half-maximal inhibitory concentration
miRs: microRNA
MMP-9: Matrix metalloproteinase-9
MRI: Magnetic resonance imaging
Mut: Mutant
OLAP: Olaparib
PARP: Poly(ADP-ribose) polymerase
PARPi: Poly(ADP-ribose) polymerase inhibitor
PDAC: Pancreatic ductal adenocarcinoma
PD-L1: Programmed death-ligand 1
PDOs: Patient-derived organoids
P-gp: P-glycoprotein
RRM1: Ribonucleotide reductase catalytic subunit M1
RRM2: Ribonucleotide reductase regulatory subunit M2
SRB: Sulforhodamine B
TS: Thymidylate synthase
Wt: Wild-type
