# Supplementary material for: A first-in-class inhibitor of homologous recombination DNA repair counteracts tumour growth, metastasis and therapeutic resistance in pancreatic cancer
Source: J Exp Clin Cancer Res. 2025 Apr 24;44:129. doi: 10.1186/s13046-025-03389-5 (PMC12020112; doi:10.1186/s13046-025-03389-5)
Supplement: Supplementary file 1 — Supplementary Material 1 [file 13046_2025_3389_MOESM1_ESM.docx]

**Supplementary material**

**A first-in-class inhibitor of homologous recombination DNA repair counteracts tumour growth, metastasis and therapeutic resistance in pancreatic cancer**

Juliana Calheiros, Rita Silva, Filipa Barbosa, João Morais, Sara Reis Moura, Sofia Almeida, Elena Fiorini, Silva Mulhovo, Tatiana Q. Aguiar, Tao Wang, Sara Ricardo, Maria Inês Almeida, Lucília Domingues, Sonia A. Melo, Vincenzo Corbo, Maria José U. Ferreira, Lucília Saraiva

**Table S1.** Characterization of human immortalized PDAC cells.

| **Cell line** | **RRID** | **Tissue/disease** | **p53 status** | **KRAS status** | **BRCA status** | **Supplier** |
| --- | --- | --- | --- | --- | --- | --- |
| **PANC-1** | [RRID:CVCL_0480](https://www.cellosaurus.org/CVCL_0480) | Pancreatic ductal adenocarcinoma | Homozygous R273H | Heterozygous G12D | Wild-type | ATCC (Rockville, MD, USA) |
| **GEM-resistant and parental MIA-PaCa-2** | [RRID:CVCL_0428](https://www.cellosaurus.org/CVCL_0428) |  | Homozygous R248W | Homozygous G12C | Wild-type | Provided by Professor Luigi Sapio from Università degli Studi della Campania,  Italy |
| **MIA-PaCa-2** |  |  |  |  |  | ATCC (Rockville, MD, USA) |
| **AsPC1** | [RRID:CVCL_0152](https://www.cellosaurus.org/CVCL_0152) |  | Heterozygous  C135A | Homozygous G12D | Wild-type |  |
| **BxPC3** | [RRID:CVCL_0186](https://www.cellosaurus.org/CVCL_0186) |  | Homozygous  Y220C | Wild-type | Wild-type |  |
| **Hs766T** | [RRID:CVCL_0334](https://www.cellosaurus.org/CVCL_0334) |  | Wild-type | Homozygous  Q61H | Wild-type |  |
| **HPAF-II** | [RRID:CVCL_0313](https://www.cellosaurus.org/CVCL_0313) |  | Homozygous  P151S | Heterozygous G12D | Wild-type |  |
| **Capan-1** | [RRID:CVCL_0237](https://www.cellosaurus.org/CVCL_0237) |  | Homozygous A159V | Homozygous G12V | Hemizygous 6174delT mutBRCA2 | Provided by Professor Bruno Costa-Silva from Champalimaud Foundation, Lisbon, Portugal |

**Table S2.** List of antibodies used in western blot (WB), immunofluorescence (IF) and immunohistochemistry (IHC).

| Antigen | Blocking solution (WB, IF) or antigen retrieval buffer (IHC) | Dilution | Application | Supplier | Cat# / RRID |
| --- | --- | --- | --- | --- | --- |
| Primary antibodies | | | | | |
| GAPDH (6C5) mouse mAb | 5 % (w/v) skimmed milk | 1:10000 | WB | Santa Cruz Biotechnology | [Cat# sc-32233, RRID:AB_627679](http://antibodyregistry.org/AB_627679) |
| Vinculin (7F9) mouse mAb |  | 1:500 |  |  | [Cat# sc-73614, RRID:AB_1131294](http://antibodyregistry.org/AB_1131294) |
| RAD50 (G-2) mouse mAb |  | 1:200 |  |  | [Cat# sc-74460, RRID:AB_1128909](http://antibodyregistry.org/AB_1128909) |
| RAD52 (F-7) mouse mAb |  |  |  |  | [Cat# sc-365341, RRID:AB_10851346](http://antibodyregistry.org/AB_10851346) |
| RAD54 (F-11) mouse mAb |  |  |  |  | Cat# [sc-374598, RRID:AB_10989787](http://antibodyregistry.org/AB_10989787) |
| PUMA (G-3) mouse mAb |  |  |  |  | Cat# [sc-374223, RRID:AB_10987708](http://antibodyregistry.org/AB_10987708) |
| CDC20 (E-7) mouse mAb |  |  |  |  | [Cat# sc-13162, RRID:AB_628089](http://antibodyregistry.org/AB_628089) |
| CDC25c (H-6) mouse mAb |  |  |  |  | [Cat# sc-13138, RRID:AB_627227](http://antibodyregistry.org/AB_627227) |
| ENT1 (F-12) mouse mAb |  |  |  |  | [Cat# sc-377283](https://www.scbt.com/pt/p/ent1-antibody-f-12?srsltid=AfmBOoqwUah9fy15H69mTkRwYCL7WLvKngmYv4gvXpEQKqX2CNDO-g5C) |
| R2 (A-5) mouse mAb |  |  |  |  | [Cat# sc-398294, RRID:AB_2894824](http://antibodyregistry.org/AB_2894824) |
| β-catenin (E-5) mouse mAb |  |  |  |  | [Cat# sc-7963, RRID:AB_626807](http://antibodyregistry.org/AB_626807) |
| Zeb1 (H-3) mouse mAb |  |  |  |  | [Cat# sc-515797, RRID:AB_2934316](http://antibodyregistry.org/AB_2934316) |
| E-cadherin (G-10) mouse mAb | 10 mM sodium citrate (pH 6.0) | 1:100 | IHC |  | [Cat# sc-8426, RRID:AB_626780](http://antibodyregistry.org/AB_626780) |
|  | 5 % (w/v) skimmed milk |  | WB |  |  |
| p21 (F-5) mouse mAb |  |  |  |  | [Cat# sc-6246, RRID:AB_628073](http://antibodyregistry.org/AB_628073) |
| BARD1 (E-11) mouse mAb |  | 1:500 |  |  | [Cat# sc-74559, RRID:AB_2061237](http://antibodyregistry.org/AB_2061237) |
| BAX (B-9) mouse mAb | 10 mM sodium citrate (pH 6.0) | 1:100 | IHC |  | [Cat# sc-7480, RRID:AB_626729](http://antibodyregistry.org/AB_626729) |
| Ki-67 (SP6) rabbit mAb |  | 1:200 |  | Thermo Fisher Scientific | [Cat# MA5-14520, RRID:AB_10979488](http://antibodyregistry.org/AB_10979488) |
| MUC1 (HMFG2) mouse mAb | 10 mM sodium citrate (pH 6.0) | 1:100 | IHC | Merck Millipore | Cat# MABC1613 |
| γH2AX (phospho-Ser139) rabbit pAb | 5 % (w/v) bovine serum albumin | 1:10000 | WB | Abcam | [Cat# ab2893, RRID:AB_303388](http://antibodyregistry.org/AB_303388) |
|  | 5 % (w/v) bovine serum albumin | 1:3000 | IF |  |  |
|  | 10 mM sodium citrate (pH 6.0) | 1:1000 | IHC |  |  |
| Survivin rabbit mAb | Tris/EDTA buffer (pH 9.0) | 1:250 | IHC |  | [Cat# ab76424, RRID:AB_1524459](http://antibodyregistry.org/AB_1524459) |
|  | 5 % (w/v) skimmed milk | 1:5000 | WB |  |  |
| RAD51 [EPR4030(3)] rabbit mAb |  | 1:500 |  |  | [Cat# ab133534, RRID:AB_2722613](http://antibodyregistry.org/AB_2722613) |
|  | 5 % (w/v) bovine serum albumin | 1:800 | IF |  |  |
|  | Tris/EDTA buffer (pH 9.0) | 1:400 | IHC |  |  |
| BRCA1 [MS110] mouse mAb | 5 % (w/v) bovine serum albumin | 1:200 | IF |  | [Cat# ab16780, RRID:AB_2259338](http://antibodyregistry.org/AB_2259338) |
|  | 10 mM sodium citrate (pH 6.0) | 1:200 | IHC |  |  |
| MMP-9 mouse mAb | 5 % (w/v) skimmed milk | 1:800 | WB |  | [Cat# ab58803, RRID:AB_944235](http://antibodyregistry.org/AB_944235) |
|  | Tris/EDTA buffer (pH 9.0) | 1:200 | IHC |  |  |
| PD-L1/CD274 mouse mAb | Tris/EDTA buffer (pH 9.0) | 1:5000 | IHC | Proteintech | [Cat# 66248-1-Ig, RRID:AB_2756526](http://antibodyregistry.org/AB_2756526) |
| α-SMA rabbit pAb |  | 1:7000 |  |  | [Cat# 55135-1-AP, RRID:AB_10949628](http://antibodyregistry.org/AB_10949628) |
| COL11A1 rabbit pAb |  | 1:700 |  |  | [Cat# 21841-1-AP, RRID:AB_2918074](http://antibodyregistry.org/AB_2918074) |
| BRCA1 (A8X9F) rabbit mAb | 5 % (w/v) skimmed milk | 1:1000 | WB | Cell signaling | [Cat# 14823, RRID:AB_2798631](http://antibodyregistry.org/AB_2798631) |
| BRCA2 (D9S6V) rabbit mAb |  | 1:800 |  |  | [Cat# 10741, RRID:AB_2797730](http://antibodyregistry.org/AB_2797730) |
| PARP-1 rabbit pAb |  | 1:1000 |  |  | [Cat# 9542, RRID:AB_2160739](http://antibodyregistry.org/AB_2160739) |
| MDR1/ABCB1 (D3H1Q) rabbit mAb | 5 % (w/v) bovine serum albumin | 1:1000 |  |  | [Cat# 12683, RRID:AB_2715689](http://antibodyregistry.org/AB_2715689) |
| Ku80 (C48E7) rabbit mAb | 10 mM sodium citrate (pH 6.0) | 1:400 | IHC |  | [Cat# 2180, RRID:AB_2218736](http://antibodyregistry.org/AB_2218736) |
| Secondary antibodies | | | | | |
| Anti-mouse HRP-conjugated | 5 % (w/v) skimmed milk | 1:10000 | WB | Abcam | [Cat# ab6789, RRID:AB_955439](http://antibodyregistry.org/AB_955439) |
| Anti-rabbit HRP-conjugated |  |  |  |  | [Cat# ab6721, RRID:AB_955447](http://antibodyregistry.org/AB_955447) |
| Anti-mouse alexa fluor 488-conjugated | 1 % (w/v) bovine serum albumin | 1:200 | IF | Thermo Fisher Scientific | [Cat# A-11001, RRID:AB_2534069](http://antibodyregistry.org/AB_2534069) |
| Anti-rabbit alexa fluor 488-conjugated |  |  |  |  | [Cat# A-11008, RRID:AB_143165](http://antibodyregistry.org/AB_143165) |


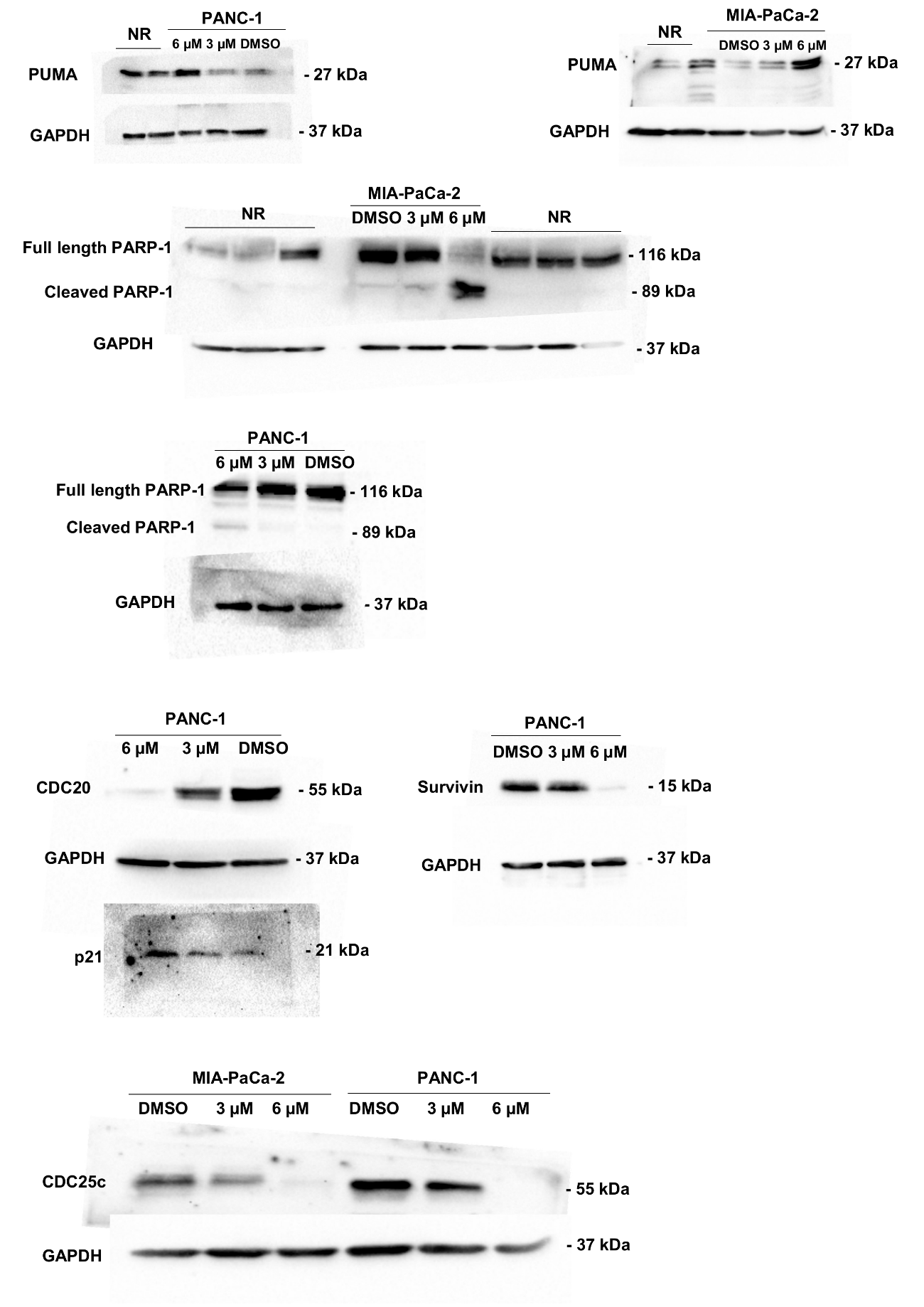


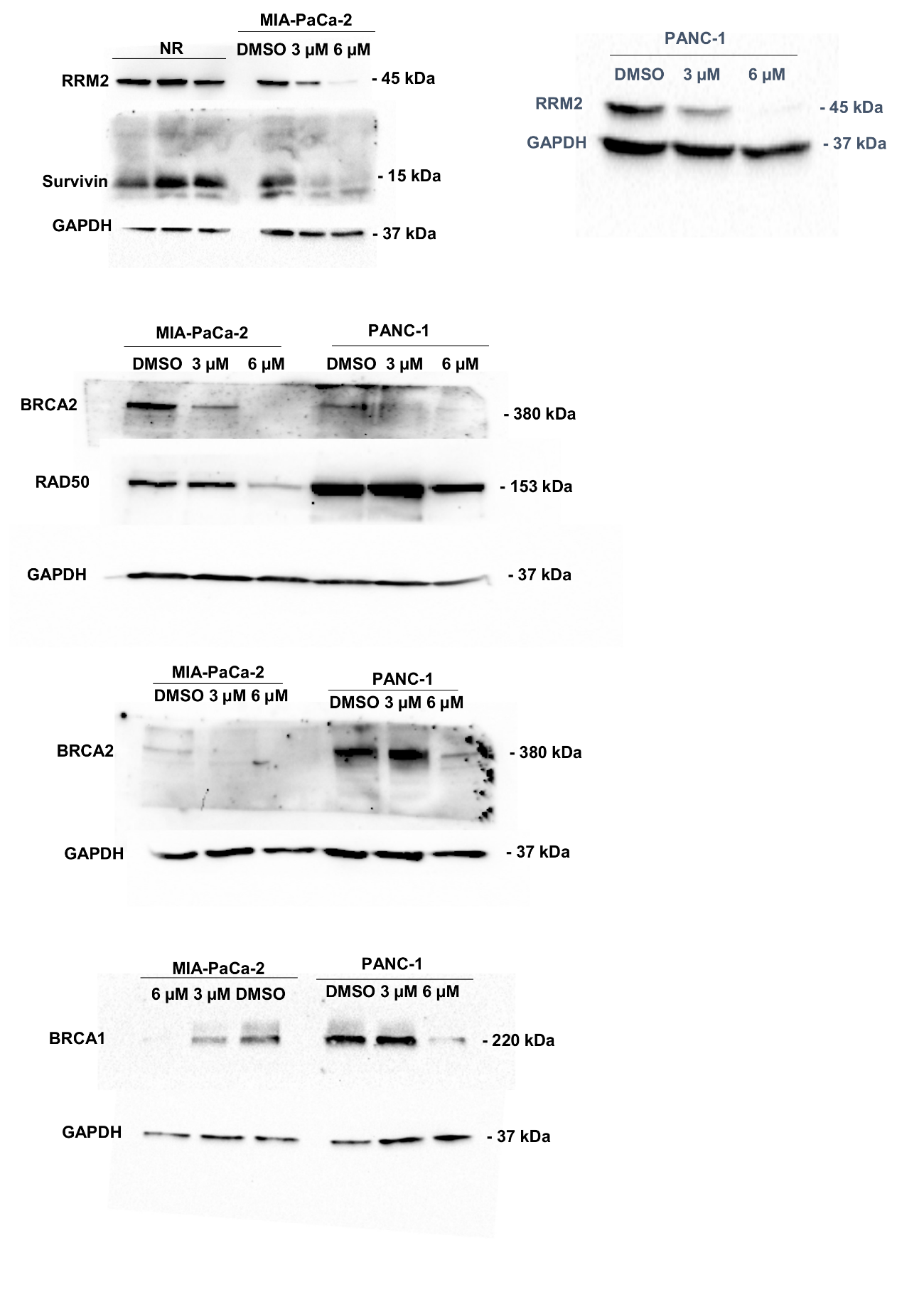

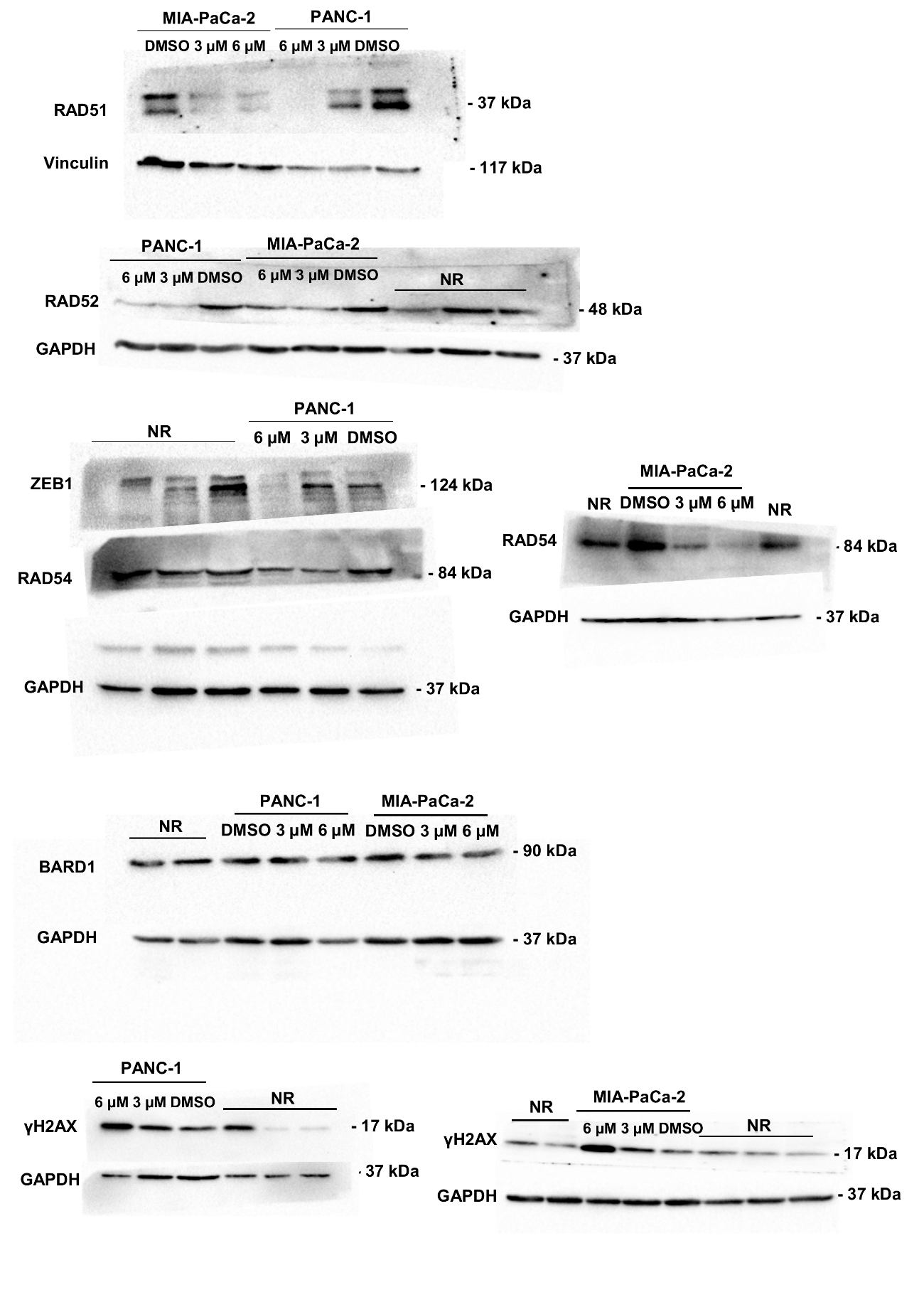

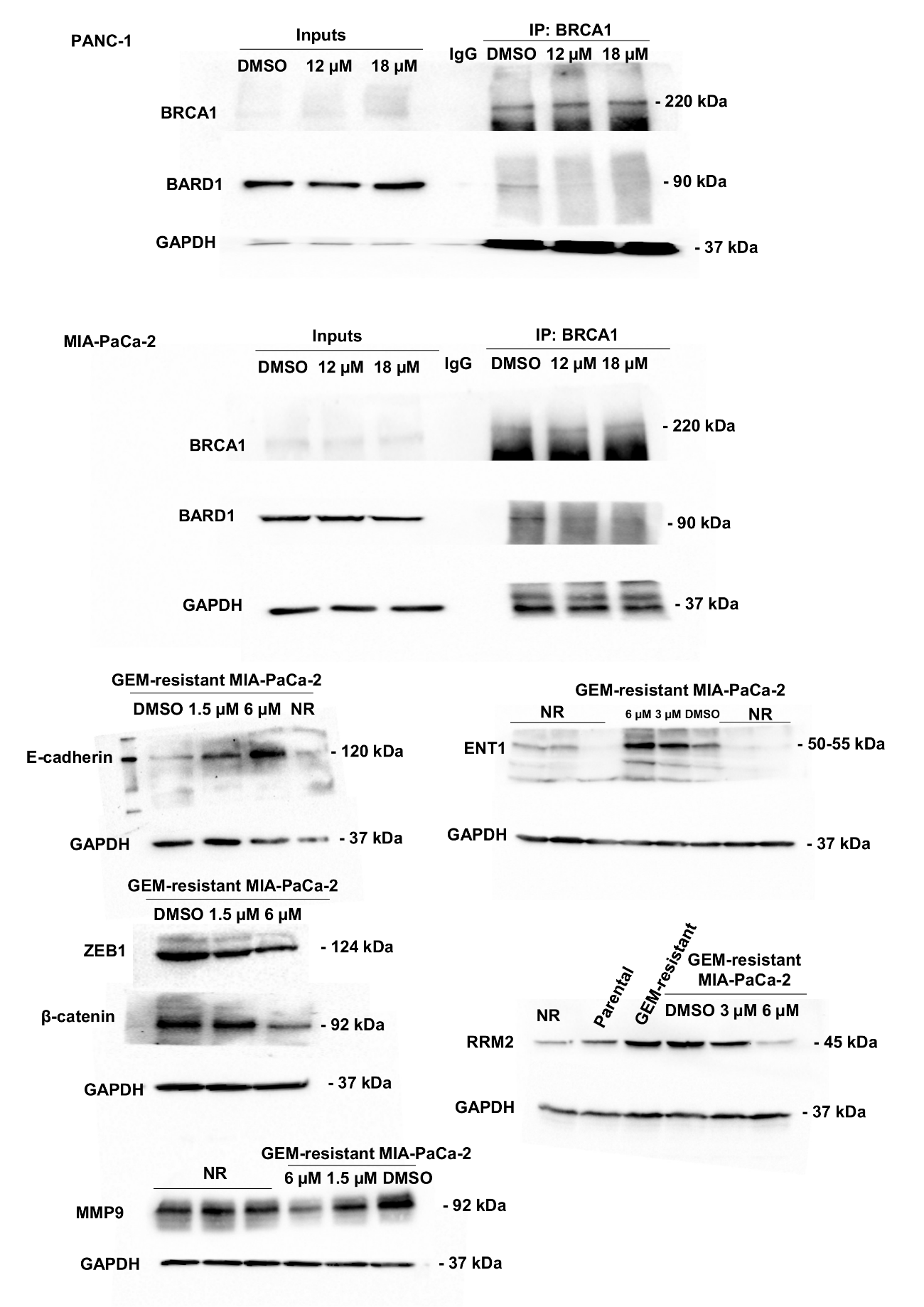

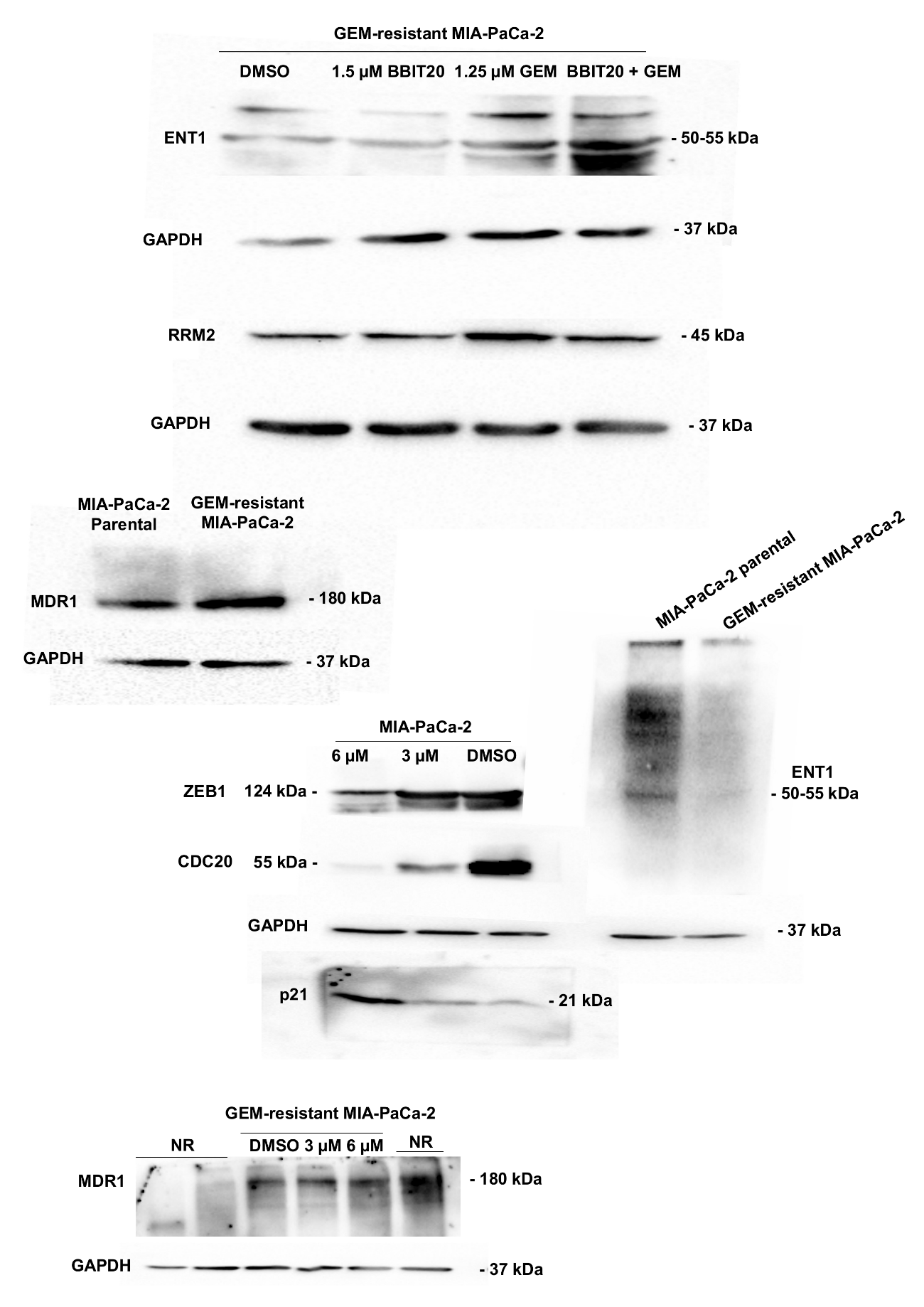


**Figure S1.** Whole blot images. NR indicates samples of other non-related work.


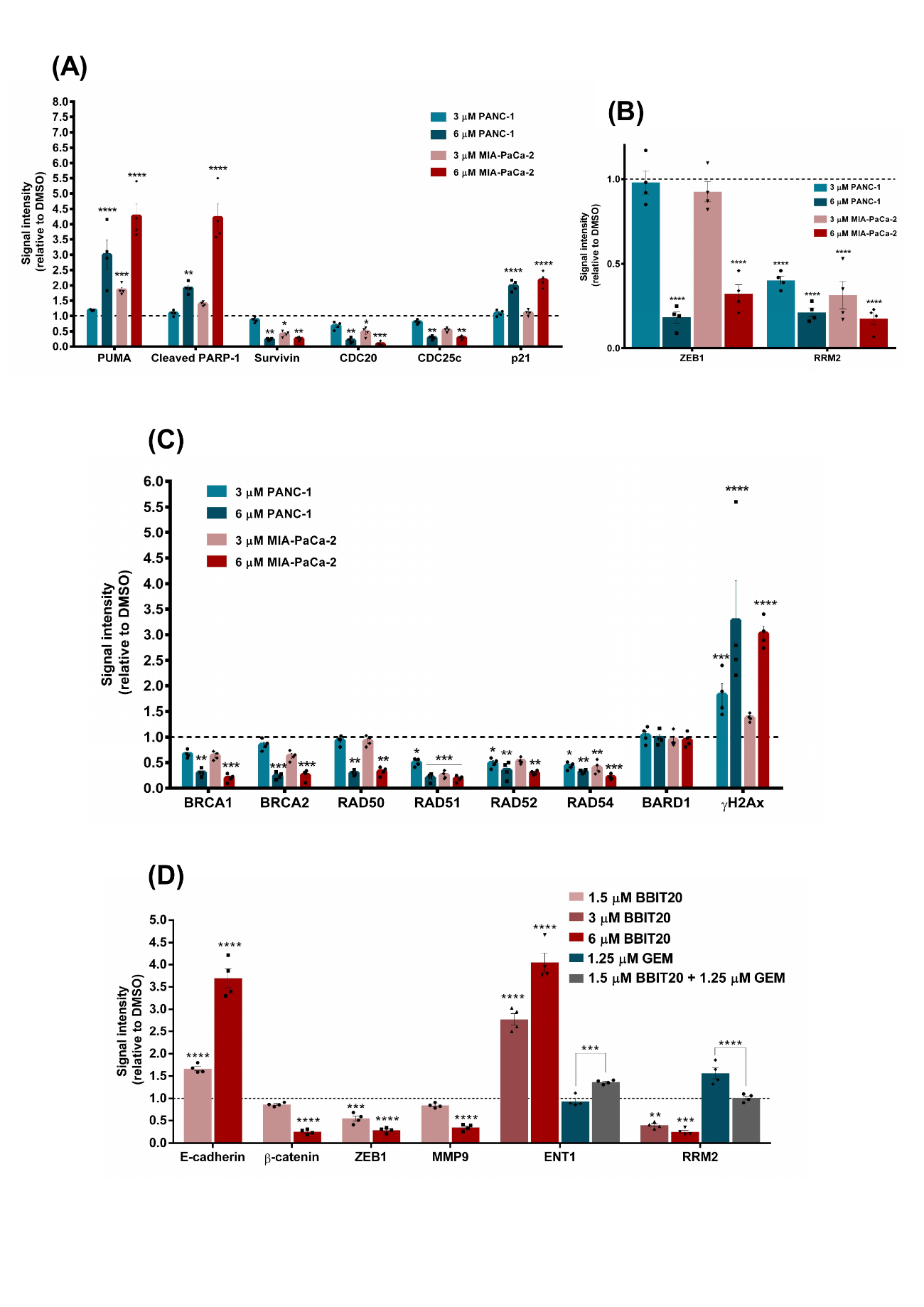
**Figure S2.** **Quantification of protein expression levels.** In (A), protein expression levels of PUMA, cleaved PARP-1, survivin, CDC20, CDC25c and p21, in PANC-1 and MIA-PaCa-2 cells, treated with 3 and 6 µM of BBIT20 for 48 h. In (B), protein expression levels of ZEB1, in PANC-1 and MIA-PaCa-2 cells treated with 3 and 6 µM of BBIT20 for 24 h, and RRM2, in PANC-1 and MIA-PaCa-2 cells treated with 3 and 6 µM of BBIT20 for 24 h and 48 h, respectively. In (C), protein expression levels of BRCA1, BRCA2, RAD50, RAD51, RAD52, RAD54, BARD1 and γH2AX, in PANC-1 and MIA-PaCa-2 cells, treated with 3 and 6 µM of BBIT20 for 48 h. In (D), protein expression levels of E-cadherin, β-catenin, ZEB1 and MMP-9, in GEM-resistant MIA-PaCa-2 cells, treated with 1.5 and 6 µM of BBIT20, for 48 h; ENT1 and RRM2 protein levels, in GEM-resistant MIA-PaCa-2 cells, treated with 3 and 6 µM of BBIT20, for 24 h; and ENT1 and RRM2 protein levels, in GEM-resistant MIA-PaCa-2 cells, treated with 1.25 µM of GEM or 1.25 µM of GEM in combination with 1.5 µM of BBIT20, for 48 h. Signal intensity of DMSO was set as 1. Data are mean ± SEM of four independent experiments; values significantly different from DMSO or GEM: **p*<0.05, ***p*<0.01, ****p*<0.001, *****p*<0.0001 (two-way ANOVA with Dunnett’s test).

**Table S3.** Characterization of patient-derived PDAC organoids.

| **Patient-derived organoid** | ***KRAS*** | ***TP53*** | ***CDKN2A*** | ***SMAD4*** | ***BRCA*** |
| --- | --- | --- | --- | --- | --- |
| **1** | G12D | Missense variant Y234C | Frameshift variant RL102-103X / loss | Loss-of-heterozygosity | Wild-type |
| **2** | G12D | Missense variant I195T | Stop gained R58* | Missense variant A406V / loss | Wild-type |
| **3** | G12D | Missense variant G245S | Homozygous deletion | Missense variant G365D | Wild-type |


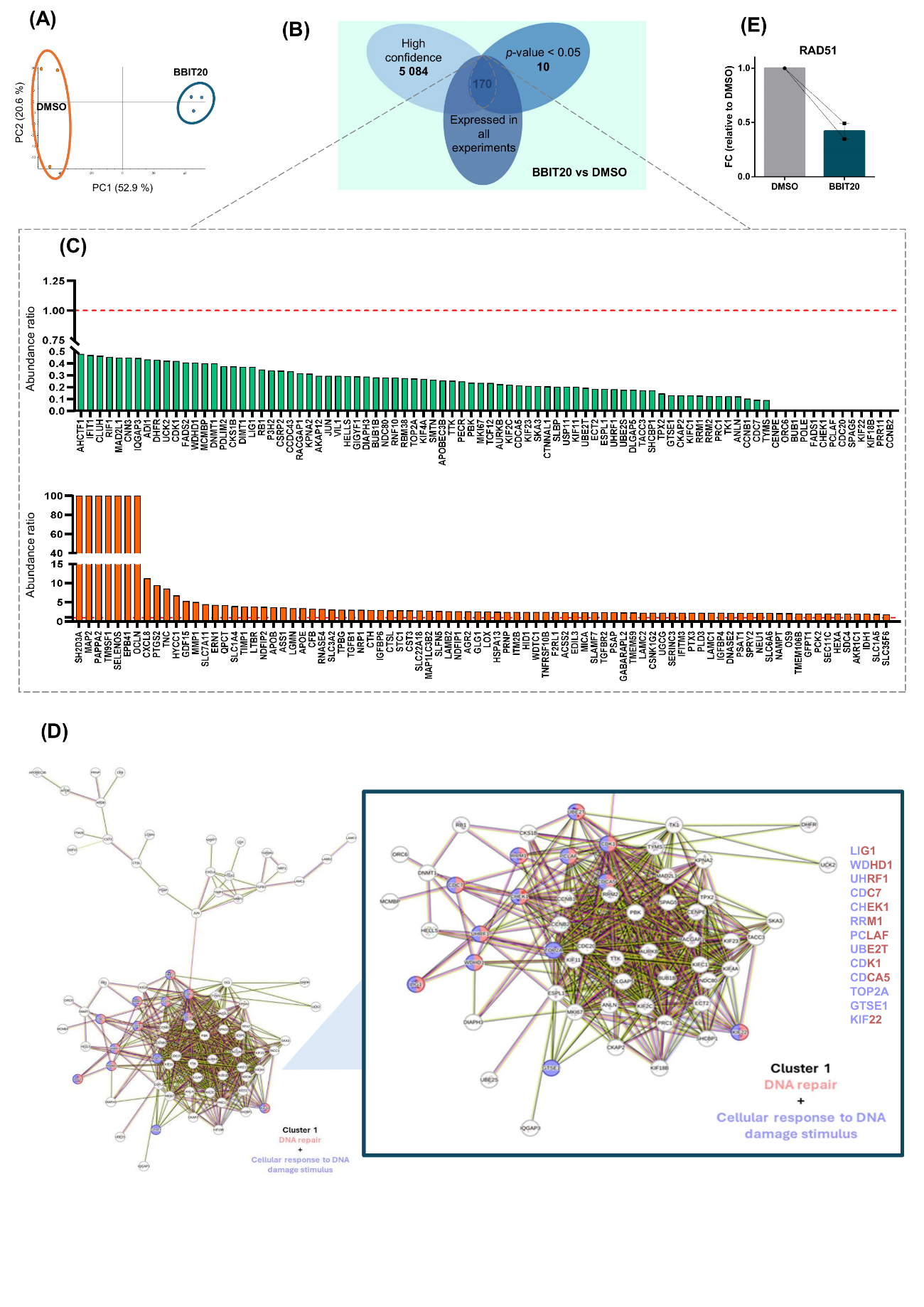
**Figure S3. Proteomic analysis of BBIT20 downstream targets and molecular pathways in MIA-PaCa-2 cells, after 48 h of treatment.** In (A), principal component analysis (PCA) plot shows the clustering of the three independent experiments after normalization. Only proteins with ≥ 2 unique peptides were considered, following contaminant removal (Proteome Discoverer); in (B), Venn diagram with the differentially expressed proteins (*p*-value < 0.05), including only those with high confidence false discovery rate (FDR) and consistent expression trends across independent experiments. A total of 5 265 proteins were detected with ≥ 2 unique peptides after contaminant removal. In (C), bar graph representing the number of differentially downregulated (green bars) and upregulated (orange bars) proteins (*p*-value < 0.05), including those expressed in all experiments with high confidence FDR and consistent expression trends across independent experiments. In (D), STRING analysis demonstrating the network of proteins related to DNA repair and cellular responses to DNA damage stimulus modulated by BBIT20. Representation of protein-protein interaction network of differentially expressed proteins (*p*-value < 0.05) using STRING and a higher confidence score (0.700). Four clusters were identified using a k-means clustering. Representation of cluster 1 highlighting DNA repair (red) and cellular response to DNA damage stimulus (blue). In (E), fold-change (FC) relative to DMSO for the RAD51 expression in MIAPaCa-2 cells treated with 6 µM of BBIT20 for 48 h of two independent experiments.


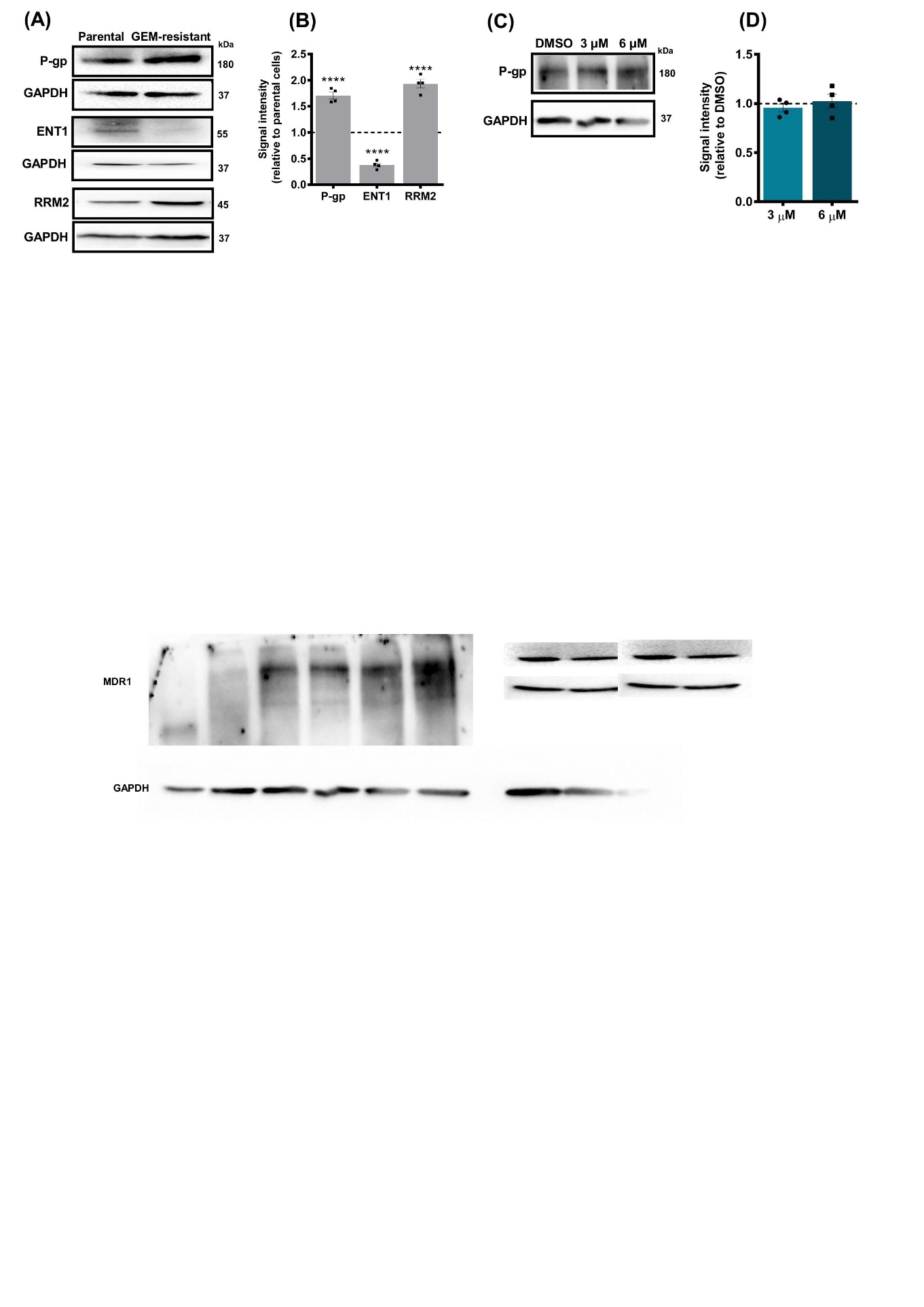


**Figure S4. Molecular alterations in GEM-resistant MIA-PaCa-2 cells: upregulation of P-gp and RRM2 and downregulation of ENT1 expression levels. BBIT20 does not interfere with P-gp protein levels, in GEM-resistant MIA-PaCa-2 cells.** In (A, B), protein expression levels of P-glycoprotein (P-gp), equilibrative nucleoside transporter 1 (ENT1) and ribonucleotide reductase regulatory subunit M2 (RRM2) in parental and GEM-resistant MIA-PaCa-2 cells. In (A), representative blots are shown, GAPDH was used as a loading control. In (B), quantification of protein levels, using signal of parental cells set as 1; data are mean ± SEM of four independent experiments; values significantly different from parental cells: *****p*<0.0001 (two-way ANOVA with Sidak’s test). In (C, D), P-gp expression in GEM-resistant MIA-PaCa-2 cells treated with 3 and 6 µM of BBIT20 for 48 h. In (C), representative blots are shown, GAPDH was used as a loading control. In (D), quantification of P-gp protein levels, using values of DMSO set as 1; data are mean ± SEM of four independent experiments; values not significantly different from DMSO: *p*>0.05 (one-way ANOVA with Dunnett’s test).


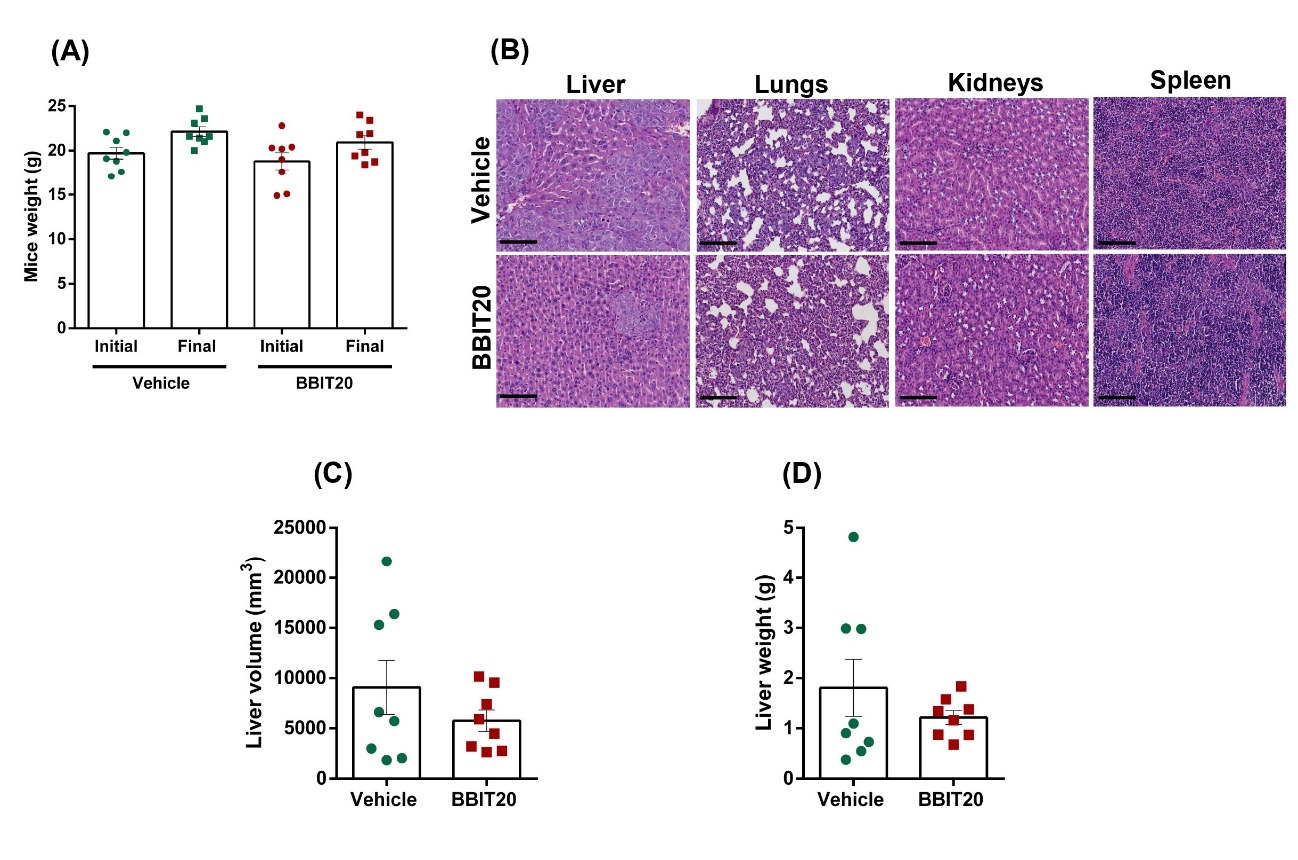


**Figure S5.** **Biosafety evaluation of BBIT20 in mice.** In (A), mice’s weight at the beginning of the treatments and at the final of the experiment in vehicle- and BBIT20-treated groups, represented as mean ± SEM of eight animals. Mice weight is not significantly different from vehicle: *p*>0.05 (unpaired *t*-test). In (B), H&E staining images (scale bar = 100 µm; 100× magnification) of major organs (liver, lungs, kidneys and spleen) from mice after treatment with vehicle or BBIT20. In (C, D), data from liver volume (C) and weight (D) of mice endpoint were analysed for C57BL/6 Rag2^-/-^IL2rg^-/-^ xenograft mice treated with vehicle or 2 mg/kg of BBIT20. Results are shown as mean ± SEM of eight livers; values are not significantly different from vehicle: *p* > 0.05 (unpaired student’s *t*-test).
